# Phylogeny and historical biogeography analysis support Caucasian and Mediterranean centres of origin of key holoparasitic Orobancheae (Orobanchaceae) lineages

**DOI:** 10.3897/phytokeys.174.62524

**Published:** 2021-03-12

**Authors:** Renata Piwowarczyk, Adam C. Schneider, Grzegorz Góralski, Dagmara Kwolek, Magdalena Denysenko-Bennett, Anna Burda, Karolina Ruraż, Andrzej J. Joachimiak, Óscar Sánchez Pedraja

**Affiliations:** 1 Center for Research and Conservation of Biodiversity, Department of Environmental Biology, Institute of Biology, Jan Kochanowski University, Uniwersytecka 7, PL-25-406 Kielce, Poland Jan Kochanowski University Kielce Poland; 2 Department of Biology and Health Sciences, Hendrix College, 1600 Washington Ave, Conway AR 72032, USA Department of Biology and Health Sciences, Hendrix College Conway United States of America; 3 Department of Plant Cytology and Embryology, Institute of Botany, Jagiellonian University, Gronostajowa 9, PL-30-387 Kraków, Poland Jagiellonian University Kraków Poland; 4 Grupo Botánico Cantábrico, ES-39722 Liérganes (Cantabria), Spain Grupo Botánico Cantábrico Liérganes Spain

**Keywords:** Biodiversity hotspot, chronogram, *
Cistanche
*, divergence time, historical biogeography, *
Orobanche
*, *
Phelipanche
*, *
Phelypaea
*

## Abstract

The extensive diversity of the tribe Orobancheae, the most species-rich lineage of holoparasitic Orobanchaceae, is concentrated in the Caucasus and Mediterranean regions of the Old World. This extant diversity has inspired hypotheses that these regions are also centres of origin of its key lineages, however the ability to test hypotheses has been limited by a lack of sampling and phylogenetic information about the species, especially in the Caucasus region. First, we assessed the phylogenetic relationships of several poorly known, problematic, or newly described species and host-races of four genera of Orobancheae occurring in the Caucasus region–*Cistanche*, *Phelypaea*, *Phelipanche* and *Orobanche*–using nuclear ribosomal (ITS) and plastid (*trnL–trnF*) sequence data. Then we applied a probablistic dispersal-extinction-cladogenesis model of historical biogeography across a more inclusive clade of holoparasites, to explicitly test hypotheses of Orobancheae diversification and historical biogeography shifts. In sum, we sampled 548 sequences (including 196 newly generated) from 13 genera, 140 species, and 175 taxa across 44 countries. We find that the Western Asia (particularly the Caucasus) and the Mediterranean are the centre of origin for large clades of holoparasitic Orobancheae within the last 6 million years. In the Caucasus, the centres of diversity are composed both of long-branch taxa and shallow, recently diversified clades, while Orobancheae diversity in the Mediterranean appears to represent mainly recent diversification.

## Introduction

The tribe Orobancheae is the oldest and most species-rich of the three lineages of holoparasites comprising the cosmopolitan family Orobanchaceae, with a crown age dating to the mid-Miocene (McNeal et al. 2013; Schneider and Moore 2017). In its current circumscription the Orobanchaceae includes the holoparasites that have always comprised Orobanchaceae s. str., and hemiparasites traditionally included in Scrophulariaceae (Olmstead et al. 2001; McNeal et al. 2013). In sum, this is the largest parasitic plant family with 102 genera and over 2,100 species (Nickrent 2020) which together with its variety of trophic modes makes it a valuable model for studying the evolution and physiology of parasitism (Westwood et al. 2010).

The Mediterranean Basin and Caucasus region of western Asia are centres of extant diversity for the two most diverse genera in the Orobancheae, *Orobanche* L. and *Phelipanche* Pomel (ca. 150 and 60 described species, respectively) (Piwowarczyk et al. 2019), and are more generally recognised as one of the world’s hotspots of biodiversity (Mittermeier et al. 2005). Recent taxonomic and field studies in the Caucasus have helped clarify the nomenclature, taxonomy, and distribution of taxa from four genera (*Orobanche* L., *Phelipanche* Pomel, *Phelypaea* L. (= *Diphelypaea* Nicolson) and *Cistanche* Hoffmannsegg & Link), and revealed many endemic and host-specific species in this region that had previously been overlooked (e.g., Piwowarczyk 2015; Piwowarczyk et al. 2015, 2017a, b, c, d, 2018a, b, c, 2019, 2020a, 2021). Other researchers have refined the understanding of these four genera in the Mediterranean Basin, as well as the monotypic *Boulardia* F.W. Schultz. (e.g., Foley 2001; Carlón et al. 2003, 2005, 2008; Domina and Arrigoni 2007; Jeanmonod and Habashi 2007; Pujadas Salvà 2009; Domina et al. 2011, 2013; Frajman et al. 2013). Together, the Mediterranean and the Caucasus have been hypothesised as refugia for both plant and animal lineages during the Pleistocene ice ages (Taberlet et al. 1998; Hewitt 1999; Lumibao et al. 2017), and some authors even propose the Caucasus together with the Middle East and Central Asian high mountains as the main area of origin of Old World broomrapes (*Orobanche* and *Phelipanche*, Rätzel and Uhlich 2004).

While regions of high extant diversity for any lineage may be the result of *in situ* diversification, this is not necessarily the case. Thus, hypotheses of historical biogeography must be explicitly tested. Schneider and Moore (2017) used a statistical phylogenetic framework to infer the divergence times and historical biogeography of the Orobancheae to the extent possible given the limitations of a depauperate fossil record and the increased rates of molecular evolution that are characteristic of parasitic plants (Bromham et al. 2013). While an important first step, their study focused on New World taxa and therefore lacked the taxonomic sampling or granularity of geographical data to evaluate biogeographical patterns within the Old World.

The aims of this study were two-fold. First, we sought to assess previously unknown phylogenetic relationships of Caucasian Orobancheae using nuclear ribosomal (ITS region) and plastid (*trnL–trnF*) DNA sequences. Second, we sought to evaluate the historical biogeography of Old World Orobancheae using a probabilistic dispersal-extinction-cladogenesis (DEC) model. In particular, we wanted to evaluate the hypothesis of Western Asia (especially the Caucasus) and the Mediterranean as potential refugia and/or centres of origin for major species-rich clade in the Orobancheae.

## Materials and methods

### Taxonomic sampling and data collection

For the initial phylogenetic analysis, we studied Caucasian species of *Cistanche*, *Phelypaea*, *Phelipanche* and *Orobanche*, mainly collected from Georgia, Armenia, Azerbaijan and Russia between 2014 and 2019. Specimens of some species were collected in other countries or taken from herbaria (B, ERCB, HMMNH, IRKU, KTC, LE, MW, herb. Ó. Sánchez Pedraja), or sequences were downloaded from GenBank. In total, 13 genera, 175 taxa representing 140 species (548 sequences, including 196 as new), from 44 countries, were analysed (see Suppl. material 1: Table S1). For the majority of samples, hosts were precisely identified. We assessed infraspecific variation by sampling more than one individual, often from different localities and host species. Voucher information, as well as geographic origin or GenBank accession numbers are listed in Suppl. material 1: Table S1. Newly collected plant specimens were deposited in KTC, ERCB (herbarium codes according to Index Herbariorum, Thiers 2017). Systematic division was adopted according to Beck (1930) and Teryokhin et al. (1993), the scheme followed explicitly or implicitly by most researchers, and some recent taxonomic changes made by Piwowarczyk et al. (2017a, 2018d, 2019) (Fig. 1).

**Figure 1.**
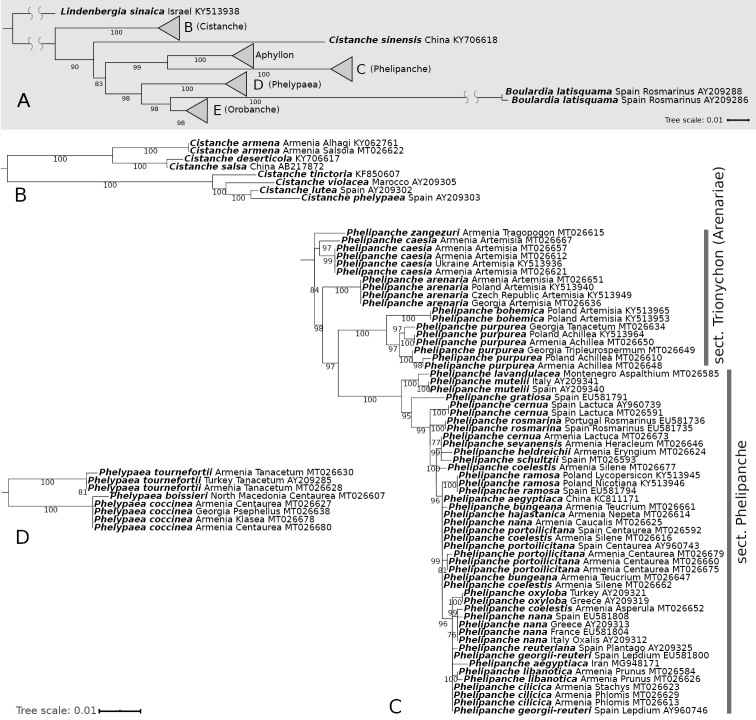
Rooted Maximum Likelihood phylogenetic tree constructed using ITS sequences. Numbers near branches show ultrafast bootstrap values (values ≥ 75 are shown). The bar represents the amount of genetic change (nucleotide substitutions per site) **A** summary of backbone (generic) relationships, branches connecting the outgroup *Lindenbergia* and *Boulardia* are shortened to fit the figure **B–E** relationships of taxa within the genera *Cistanche*, *Phelipanche*, *Phelypaea*, and *Orobanche* respectively. Species names, the country of origin, host species (if available) and GenBank number are included on the phylogeny tip labels.

**Figure 1.**
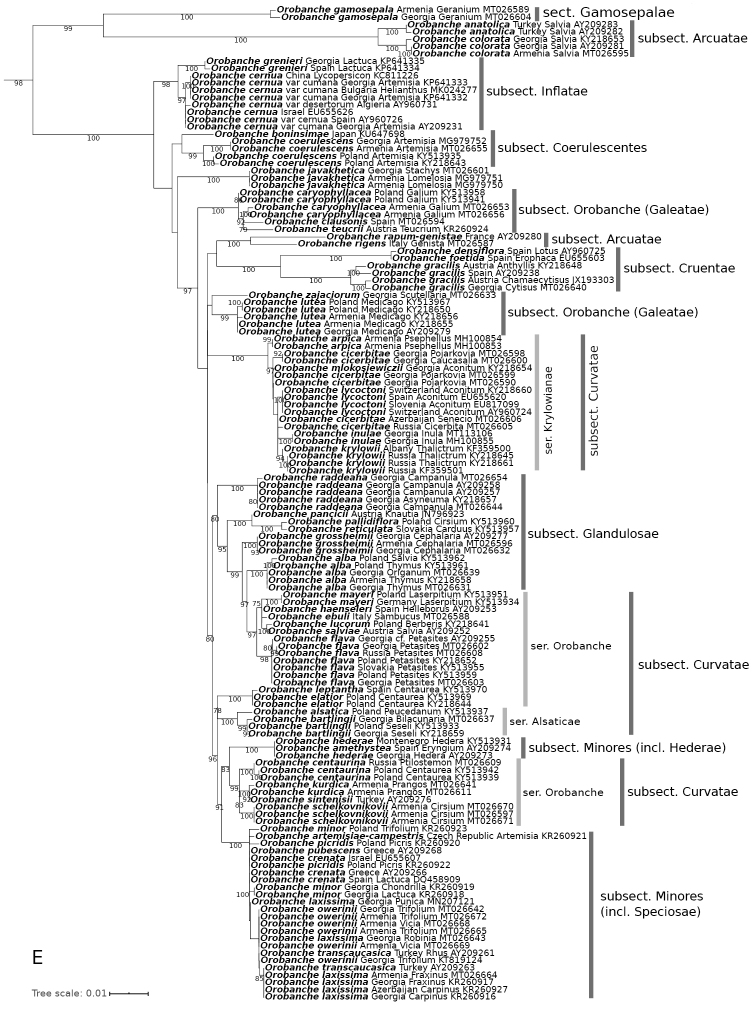
Continued.

Material used for DNA extraction was freshly collected and silica gel-dried or was obtained from herbarium vouchers. For phylogenetic studies we used two types of sequences: nuclear ITS region (internal transcribed spacer 1, 5.8S ribosomal RNA gene, internal transcribed spacer 2, later referred to as ITS) and plastid *trnL–trnF* sequence (RNA-Leu (*trnL*) intron, the partial *trnL* gene, and the intergenic spacer between the *trnL* 3’ exon and tRNA-Phe (*trnF*) gene region’s plastid DNA). These two regions are commonly used for species-level phylogenetic inference, including in the Orobancheae (ITS: Schneeweiss et al. 2004; Carlón et al. 2005, 2008; Park et al. 2008; Schneider et al. 2016; Fu et al. 2017; Piwowarczyk et al. 2018d; *trnL–trnF*: Schneider et al. 2016; Piwowarczyk et al. 2018d). DNA extraction and sequence amplification procedures follow the methods of Piwowarczyk et al. (2018d).

### Phylogenetic inference

Sequences were aligned with MAFFT v7.407 (Katoh and Standley 2013), manually corrected and trimmed. The final number of sequences and length of alignments were: for ITS 229 sequences of 671 positions, in the case of *trnL–trnF* 153 sequences, 1,337 positions long. Separate ITS and *trnL–trnF* trees were inferred instead of concatenating them into a single analysis for two main reasons: first, although preliminary trees inferred from each sequence were generally congruent, certain species did show conflicting placements (described below), perhaps due to differences in plastid versus nuclear inheritance. Second, the ITS tree is much richer in samples, because of greater availability in GenBank. Information about sequences (newly obtained and downloaded from GenBank) used in phylogenetic analysis is presented in Suppl. material 1: Table S1.

**Figure 2.**
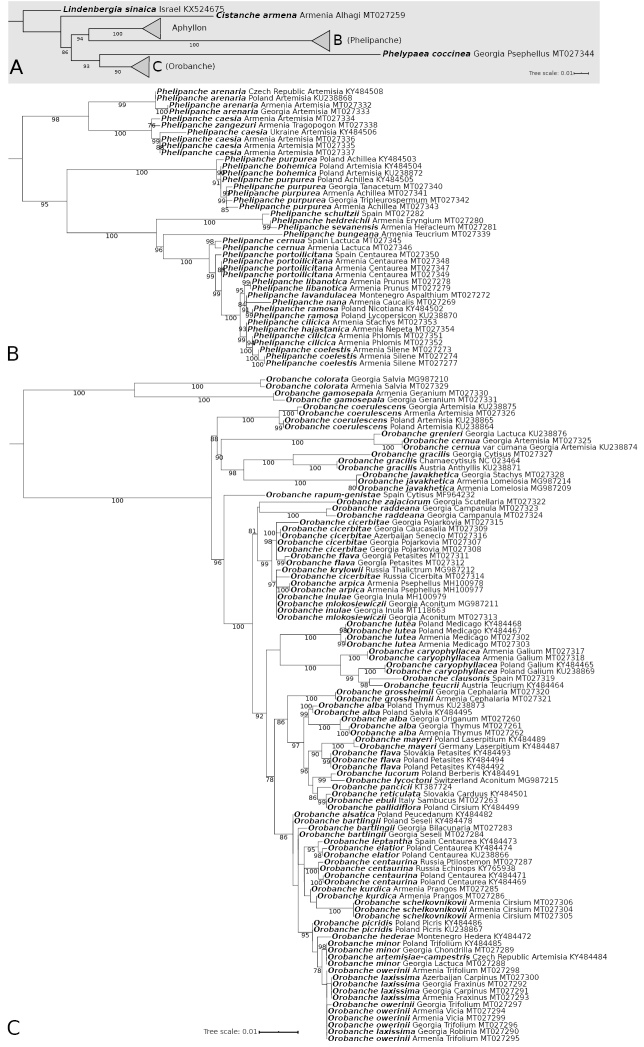
Rooted Maximum Likelihood phylogenetic tree constructed using plastid *trnL–trnF* spacer sequences. As an outgroup, *Lindenbergia sinaica* was used. Numbers near branches show ultrafast bootstrap values (values ≥ 75 are shown). The bar represents the amount of genetic change (nucleotide substitutions per site) **A** summary of backbone (generic) relationships **B***Phelipanche* clade **C***Orobanche* clade. Species names, the country of origin, host species (if available) and GenBank number are included on the phylogeny tip labels.

For both sequence alignments, Maximum Likelihood (Figs 1, 2) and Bayesian (Suppl. material 2: Fig. S1, Suppl. material 3: Fig. S2) phylogenetic trees were generated with *Lindenbergia sinaica* (Decne.) Benth. used as outgroup. Maximum Likelihood (ML) trees were calculated with IQ-TREE multicore version 1.6.12 (Nguyen et al. 2015) software, with ultrafast bootstrap approximation (2,000 bootstrap replicates). Substitution models were auto-determined by IQ-TREE using the Bayesian Information Criterion (BIC) (SYM+I+G4 for ITS and TVM+F+R3 for *trnL–trnF*). Bayesian phylogenetic trees were generated using MrBayes v. 3.2.6 (Huelsenbeck et al. 2001; Ronquist and Huelsenbeck 2003) with the following main settings: ngen = 10,000,000, samplefreq = 500, nchains = 4, checkfreq = 100,000, diagnfreq = 5,000, stopval = 0.01, stoprule = yes, relburnin = yes, burninfrac = 0.25 and, lset applyto = (all) nst = 6 rates = invgamma (for ITS) or lset applyto = (all) nst = 6 rates = gamma (for *trnL–trnF*). Substitution models according to BIC (SYM+I+G for ITS and GTR+G for *trnL–trnF*) were determined by IQ-TREE software. The trees were visualised by iTOL tool (Letunic and Bork 2016).

### Historical biogeography

To infer a chronogram for historical biogeography analysis of the tribe Orobancheae we used the ITS, PhyA, and PhyB Orobancheae alignments of Schneider and Moore (2017), improved in six ways:

1. Taxonomic coverage for *Orobanche* and *Phelipanche* was expanded based on this study.

2. Taxonomic coverage for *Cistanche* was expanded by using sequence data submitted to GenBank by Ataei (2017). Recent phylogenetic evidence support many more lineages in this genus than previously recognized (Ataei 2017; Ataei et al. 2020). Names for some of these lineages have been proposed but not yet validly published (Ataei 2017).

3. Sequences for *Gleadovia* Gamble & Prain and *Phacellanthus* Siebold & Zucc. – first published by Fu et al. (2017) and available on GenBank – were added, resulting in complete taxonomic coverage at the genus level, except for the monotypic Mexican genus *Eremitilla* Yatsk. & J.L.Contr.

4. The *trnL–trnF* plastid locus was added for most taxa based on newly generated data or pre-existing sequences (Suppl. material 1: Table S1). Although nrDNA and cpDNA partitions support conflicting relationships for a few taxa, the key nodes associated with major biogeographic transitions and discussed herein are supported by both analyses.

5. A 637 bp region of the PhyA gene was excluded from analysis because it was poorly alignable. This region appears only in our sequences for *Boschniakia himalaica* Hook. f. & Thomson ex Hook. f. and *Aphyllon ludovicianum* (Nutt.) A.Gray but not for any other species.

6. Samples for Aphyllon californicum (Cham. & Schltdl.) A.Gray subspecies feudgei, *grande*, *grayanum*, and *jepsonii* were replaced with different samples for which both ITS and *trnL–trnF* sequences were available.

Sequences matrices for each gene were aligned separately using Geneious 9.1.8 (Biomatters, Auckland, New Zealand; Kearse et al. 2012), then concatenated into a single supermatrix comprised of a 1986bp ITS + *trnL–trnF* backbone plus 3375 bp of phytochrome sequence from a subset of 20 taxa. In this case, we decided that the better branchlength estimates broadly across the tree by using multiple genes generally outweighed errors introduced for particular tips that may have conflicting ITS and *trnL–trnF* topologies. This supermatrix was used to infer a chronogram by implementing an uncorrelated lognormal relaxed clock model and a GTR+Γ substitution model in the software Revbayes v. 1.0.11 (Höhna et al. 2014). Our starting tree was generated using default parameters in RAxML-HPC v8, run on XSEDE through the CIPRES portal (Stamatakis 2014), rooted based on the results of previous comparable molecular phylogenetic studies (McNeal et al. 2013; Fu et al. 2017; Schneider and Moore 2017), and made ultrametric with a root age set to 25 (Ma) using the rate-smoothing function chronos in the R package ‘ape’ v. 5.3 (Paradis and Schliep 2019). The same divergence time calibrations and other analysis parameters were used as in Schneider and Moore (2017), except we used a new starting tree and the Markov Chain Montecarlo (MCMC) analysis was run for 4,000 iterations as a pre-burnin to tune the proposal parameters then sampled every 100 iterations for 50000 iterations with the first 15% of samples discarded as burn-in.

Each iteration consisted of 472 moves randomly scheduled from 394 possible moves. Stationarity was assessed using Tracer v.1.7.1 (Rambaut et al. 2018) and the effective sampling size of each important parameter exceeded 200: likelihood, prior, each GTR parameter and the shape parameter for the gamma distribution for each partition, speciation and extinction rates, root time, and clade ages of *Orobanche* s.l. and *Cistanche*.

**Figure 3.**
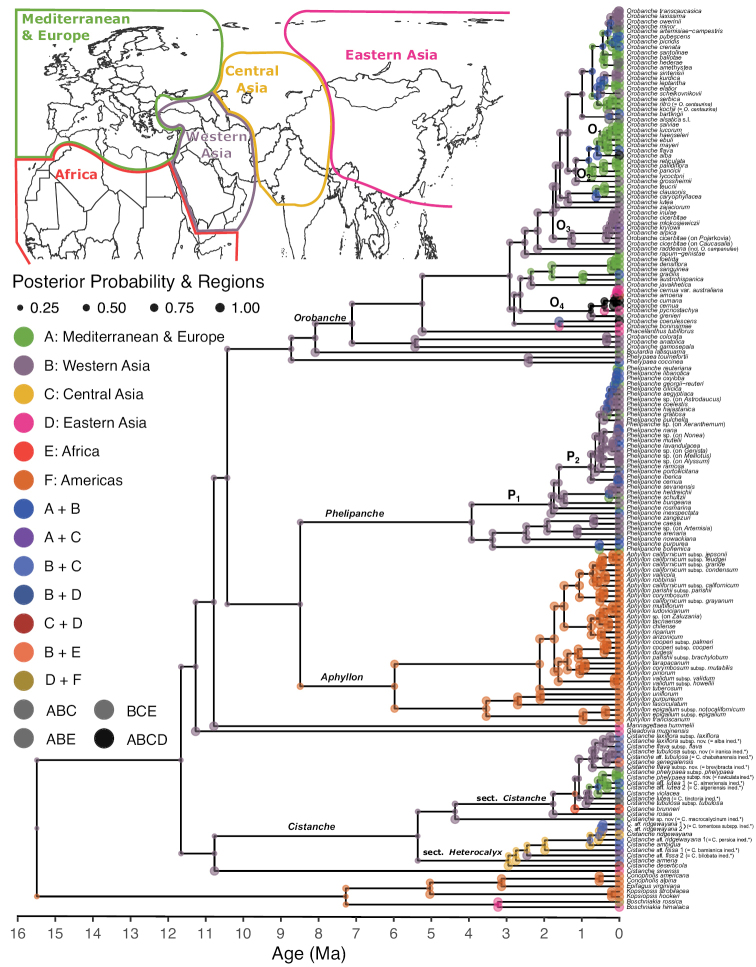
Historical biogeography of tribe Orobancheae, reconstructed using a dispersal-extinction-cladognesis model implemented in RevBayes (maximum likelihood topology, maximum clade credibility branch lengths). Coloured circles at tips represent the current biogeographical range of each sampled taxon. Circles on each node represent the reconstructed ancestral area of the most recent common ancestor of the two daughter lineages, while circles on either side of the node show the reconstructed areas immediately following cladogenesis. Circle size is proportional to posterior probability. Each colour represents a different biogeographical region or combination of regions as indicated by the map and legend to the left of the chronogram. Tip labels for *Cistanche* follow nomenclature of Ataei et al. (2020). Asterisks indicate names proposed by Ataei (2017) but not yet validly published.

For biogeographical analysis, the global range of Orobancheae was divided into six non-overlapping regions based on physical geography and natural phytogeographic divisions (Fig. 3): (1) Europe/Mediterranean, including Central, North, Eastern and Southern Europe with Mediterranean Basin (Iberian, Italian and Balkan peninsulas with northern Africa – north from Sahara Desert, and western and southern parts of the peninsula of Turkey); (2) Western Asia, which includes Anatolia in Turkey, the Arabian Peninsula, Iran, the Levant, Mesopotamia, the Sinai Peninsula, and Caucasus (with Transcaucasia); (3) Central Asia, including the area from the Caspian Sea to western China, and from Afghanistan, through Turkmenistan, Tajikistan, Uzbekistan, Kyrgyzstan, and Kazakhstan to the south to Russia (with Ciscaucasia) in the north; (4) East Asia, from central China eastward (Hong Kong, Macao, Mongolia, the Korean peninsula, Japan, and Taiwan) and including Australia for Orobanche cernua var. australiana (F. Muell.) Beck, the only taxon apparently native to that continent in our study; (5) Africa, south of the Mediterranean Basin (Saharan and sub-Saharan); and (6) the New World. In general, a taxon was not considered to inhabit a region if < 5% of its known range fell within the respective region boundary. To determine the range for individual species, we used a variety of peer-reviewed sources (e.g., Novopokrovskij and Tzvelev 1958; Wu and Raven 1998; Pusch and Günther 2009; Cullen 2010; Tzvelev 2015; Ataei 2017; Freeman et al. 2019; Piwowarczyk et al. 2019) and continually updated databases (Domina and Raab-Straube 2010; Sánchez Pedraja et al. 2016), supported by our knowledge acquired during field and herbaria research. Ataei (2017) was used to determine the distribution of undescribed *Cistanche* taxa who we follow along with Ataei et al. (2020) because they have the most comprehensive set of genetic data. However, some taxonomic and distributional ranges conflict with other recent treatments (Moreno Moral et al. 2018), highlighting the need for continued evaluation in this genus.

Ancestral geographical ranges were inferred by applying a dispersal-extinction-cladogenesis (DEC) model of historical biogeography to the maximum clade credibility (MCC) tree from the Bayesian analysis. The DEC model, also implemented in RevBayes, allows for sympatric speciation, allopatric speciation and anagenetic range expansion and contraction (Ree and Smith 2008). Two independent MCMC replicates were run for 1,000 iterations as a pre-burn-in to tune the proposal settings, then sampled every 5 iterations for 10000 iterations. Each iteration consisted of 11 moves randomly scheduled from 3 possible moves. Stationarity was also assessed using Tracer.

## Results

### Phylogenetic relationships

The most important results of our phylogenetic analyses clarified the position of many previously unsampled Caucasian species (Figs 1, 2, Suppl. material 2: Fig. S1, Suppl. material 3: Fig. S2). We also showed the phylogenetic relations of the newly described species, i.e., *Phelipanche zangezuri* Piwow. et al., *P. hajastanica* Piwow. et al., and *P. sevanensis* Piwow. et al., *O. javakhetica* Piwow. et al., *O. arpica* Piwow. et al. and *O. zajaciorum* Piwow.

Consistent with previous studies, the studied genera were each strongly supported as monophyletic (Bootstrap (BS) ≥ 90, Posterior Probability (PP) = 1.0).

#### Cistanche

ITS (*trnL–trnF* data was not available) trees show that *Cistanche armena* (K. Koch) M.V. Agab. (samples from two different hosts, *Alhagi* Gagnebin and *Salsola* L.) is closely related to *C. deserticola* Ma and *C. salsa* (C.A. Mey.) Beck (BS = 100, PP = 1.00), and with the later one it has sometimes been confused (Fig. 1, Suppl. material 2: Fig. S1).

#### Phelypaea

The three species from genus *Phelypaea*, *P. tournefortii* Desf. and *P. coccinea* (M. Bieb.) Poir. are clearly separated (BS = 100, PP = 1.00), however *P. boissieri* (Reut.) Stapf, first sequenced for this study, seems to be very similar to *P. coccinea*. Amplification of *trnL–trnF* in *Phelypaea* samples was successful only in the case of *P. coccinea*, so the above analysis was based only on ITS (Fig. 1, Suppl. material 2: Fig. S1).

#### Phelipanche

Based on ITS data *P. zangezuri* is separated from the clade of *P. caesia* (Rchb.) Soják (BS = 97, PP = 0.90) and the clade containing remain *Phelipanche* species (BS = 98, PP = 0.85). By contrast, *trnL–trnF* trees do not indicate separation of *P. zangezuri* and *P. caesia*. Rather, samples of *P. arenaria* form a sister clade to these two species, and together form a well-supported lineage (BS = 98, PP = 1.00) separated from the rest of *Phelipanche* (BS = 95, PP = 0.96) (Figs 1, 2, Suppl. material 2: Fig. S1, Suppl. material 3: Fig. S2). *P. sevanensis* is closely related to the group of *P. schultzii* (Mutel) Pomel and *P. heldreichii* (Reut.) Soják on all trees, and to *P. cernua* Pomel. on the ITS trees, (BS = 99, PP = 0.99) (Figs 1, 2, Suppl. material 2: Fig. S1, Suppl. material 3: Fig. S2). However, *P. hajastanica* is found in the group of slightly differentiable species on the ITS tree (Fig. 1), while on the *trnF-trnL* tree it is close to *P. cilicica* (Beck) Soják (BS = 99, PP = 0.92) (Fig. 2).

Our results showed the relationship of samples from different parts of the range of disjunctive species, such as *P. portoilicitana* (A. Pujadas & M.B. Crespo) Carlón et al. and *P. cernua*. Whereas *trnL–trnF* sequences of *P. cernua* places samples from Armenia and Spain are grouped in the same clade (BS = 98, PP = 0.94), on the ITS tree, the European samples are separated from Caucasian sample which is in the same clade as *P. sevanensis*, *P. schultzii* and *P. heldreichii* (BS = 99, PP = 0.99). Also, *P. portoilicitana*, both on ITS and *trnL–trnF* trees, show differences between samples from Armenia and Spain (Figs 1, 2, Suppl. material 3: Fig. S2).

#### Orobanche

*Orobanche gamosepala* Reut. is genetically distinct (BS = 100, PP = 1.00) from *O. anatolica* Boiss. & Reut. ex Reut./*O. colorata* K. Koch and together these species are grouped in sister clade to the rest of *Orobanche* species (ITS: BS = 99, PP = 1.00, *trnL–trnF*: BS = 100, PP = 1.00) (Figs 1, 2, Suppl. material 2: Fig. S1, Suppl. material 3: Fig. S2).

ITS sequence data indicates that *O. cicerbitae* (Uhlich & Rätzel) Tzvelev is not closely related to *O. flava* Mart. ex F.W. Schultz, however on the *trnL–trnF* trees *O. cicerbitae* from Georgia and Azerbaijan forms a common clade with *O. flava* from Georgia (BS = 98, PP = 0.97), whereas Central European samples of *O. flava* are distant (Fig. 1, Suppl. material 2: Fig. S1).

ITS sequences (Fig. 1, Suppl. material 2: Fig. S1) of high mountain *Orobanche* species, such as *O. krylowii* Beck, *O. cicerbitae*, *O. arpica*, *O. mlokosiewiczii* Piwow. et al., *O. inulae* Novopokr. & Abramov and *O. lycoctoni* Rhiner showed that they are closely related, and form a separated clade (ITS: BS = 100, PP = 1.00, *trnL–trnF*: BS = 99, PP = 1.00) included in O. ser. Krylowianae Piwow. et al. Probably these species diverged relatively recently and can be an example of recent rapid radiation. Another interesting phenomenon is the placement of the *trnL–trnF* sequence (Fig. 2, Suppl. material 3: Fig. S2) of *O. lycoctoni* on phylogenetic trees near *O. lucorum* A. Braun ex F.W. Schultz, (BS = 99, PP = 1.00), a species distantly related to the sect. Krylowianae species.

The phylogenetic position of Caucasian endemic species with unclear affinity has also been presented, in particular those previously classified in inappropriate subsections, such as *O. schelkownikovii* Tzvel., *O. grossheimii* Novopokr., *O. raddeana* Beck, and *O. laxissima* Rätzel & Uhlich (Figs 1, 2, Suppl. material 2: Fig. S1, Suppl. material 3: Fig. S2, and discussion below).

Little within-species variation is shown among the samples from different host species taken from the following species: *O. laxissima*, *O. alba* Stephan ex Willd., *O. bartlingii* Griseb., *O. caryophyllacea* Sm., *O. cicerbitae*, *O. gracilis* Sm., *O. centaurina* Bertol., *O. minor* Sm., *O. owerinii* (Beck) Beck, *O. raddeana*, *O. schelkovnikovii*, *P. cilicica*, *P. coelestis* (Reut.) Soják, *P. purpurea* (Jacq.) Soják and *P. coccinea* (Figs 1, 2, Suppl. material 2: Fig. S1, Suppl. material 3: Fig. S2).

### Historical biogeography

We find negligible support (PP < 0.4) for any single hypothesis ancestral range of lineages older than 6 million years. However, most diversification in the Orobancheae has happened relatively recently (Tables 1, 2, Fig. 3). We focus below on *Cistanche*, *Phelipanche* and *Orobanche* because these are the three most diverse lineages in the Old World and we found relatively high support for some biogeographical patterns. *Phelypaea* probably originated in West Asia (PP = 0.46) or was more widespread in West Asia and Caucasus, Europe and the Mediterranean (PP = 0.11).

**Table 1.** Divergence times with credible intervals (95% highest probability density (HPD)) and inferred historical biogeography of selected clades. Biogeographical regions defined in Methods and Figure 3.

Clade	Crown Age (Ma)		Biogeography	
	Mean	95% HPD	Region	Posterior Prob.
Cistanche sect. Heterocalyx	3.0	2.0–4.2	Central Asia	0.28 (0.42 for clade excluding *C. deserticola*)
			Western Asia	0.14 (0.27)
* Phelypaea *	2.4	1.4–3.7	Western Asia	0.46
			Western Asia + Med/Europe	0.11
* Phelipanche *	3.9	2.8–5.5	Western Asia	0.41
			Europe/Mediterranean	0.09
			Both	0.11
*Phelipanche* clade P_1_	1.8	1.1–2.4	Western Asia	0.38
			Western Asia + Med/Europe	0.32
*Phelipanche* clade P_2_	0.753	0.52–1.1	Western Asia	0.58
			Western Asia + Europe/Med	0.34
*Orobanche* clade O_1_	0.44	0.26–0.67	Europe/Mediterranean	0.93
*Orobanche* clade O_2_	0.72	0.46–1.0	Europe/Mediterranean	0.99
*Orobanche* clade O_1_+O_2_	0.81	0.52–1.1	Western Asia + Europe/Med	0.50
			Europe/Mediterranean	0.24
*Orobanche* clade O_3_	1.27	0.75–1.8	Western Asia	0.93
*Orobanche* clade O_4_	0.75	0.40–1.1	Europe/Mediterranean + Western Asia	n/a
			+ Central Asia	0.12
			+ East Asia	0.10
			+ Both	0.27

**Table 2.** Divergence times of species or clades endemic or nearly endemic to the Caucasus region.

Species or clade, or paraphyletic group^a^	Taxa	Divergence time^a^ (Ma)	95% HPD
Clade	*Phelypaea coccinea* + *P. tournefortii*	2.4	1.4–3.7
Clade	*Orobanche anatolica* + *O. colorata* + *O. gamosepala*	5.4	3.5–7.6
Paraphyletic	*Orobanche arpica*, *O. cicerbitae* on *Caucasalia*, *O. cicerbitae*, *O. inulae*, *O. mlokosiewiczii*, *O. cicerbitae* on *Pojarkovia* (+ widespread *O. krylowii*)	1.3	0.75–1.8
Species	*Orobanche zajaciorum*	1.7	1.2–2.3
Species	*Orobanche raddeana*	2.2	1.7–3.3
Species	*Orobanche grossheimii*	1.2	0.74–1.5
Clade	*Orobanche laxissima* + *O. owerinii* + *O. transcaucasica*	0.08	0.02–0.15
Species	*Orobanche javakhetica*	2.4	1.6–3.2
Species	*Orobanche kurdica*	0.08	0.0002–0.23
Species	*Orobanche schelkovnikovii*	0.50	0.30– 0.69
Species	*Phelipanche bungeana*	1.48	0.93–2.0
Clade	*Phelipanche coelestis* + *Phelipanche* “on *Astrodaucus*”	0.15	0.05–0.26
Species	*Phelipanche hajastanica*	0.28	0.16–0.40
Clade	*Phelipanche heldreichii* + *P. sevanensis*	0.19	0.08–0.32
Species	*Phelipanche* “on *Artemisia*”	1.1	0.52–1.8
Species	*Phelipanche* “on *Genista*”	0.20	0.10–0.31
Species	*Phelipanche pulchella*	0.41	0.21–0.61
Species	*Phelipanche zangezuri*	0.68	0.33–1.1

^a^Crown age indicated for clades of <1 species and paraphyletic groups; stem age indicated for single species.

#### Cistanche

The phylogeny of *Cistanche* appears to be structured by geography, with clades of species endemic to particular areas. For example, we find weak support for a Central Asian ancestor of Cistanche sect. Heterocalyx sensu Ataei, non Beck (composed of *C. salsa*, *C. bamianica* Ataei ined. (Ataei 2017), *C. bilobata* Ataei ined. (Ataei 2017), *C. deserticola*, *C. ambigua* (Bunge) Beck (= *C. trivalvis* (Trautv.) Korsh.), *C. tomentosa* Ataei ined. (Ataei 2017), *C. ridgewayana* Aitch. & Hemsl. and *C. persica* Ataei ined. (Ataei 2017)) (PP = 0.28; 0.42 for the subclade excluding the more widespread *C. deserticola*). Several extant species such as *C. persica*, *C. tomentosa*, and *C. salsa* extend further west into the Europe/Mediterranean region; we inferred that these are the result of recent range expansions (Fig. 3).

We found support that the clade of species *C. algeriensis* Ataei ined. (Ataei 2017), *C. almeriensis* Ataei ined. (Ataei 2017), *C. phelypaea* (L.) Cout. and *C. violacea* (Desf.) Hoffmanns. & Link in Cistanche sect. Cistanche sensu Ataei (= Cistanche sect. Eucistanche Beck, p.p. max.) originated from an ancestor that was either widespread throughout the European/Mediterranean region and Western Asia (PP = 0.47), or just restricted to Europe/the Mediterranean (PP = 0.44). We also inferred a Western Asian origin for the clade of species *C. chabaharensis* Ataei ined. (Ataei 2017), *C. tubulosa* (Schenk) Hook. f., *C. senegalensis* (Reut.) Beck, *C. laxiflora* Aitch. & Hemsl., and *C. flava* (C.A. Mey.) Korsh. (PP = 0.95), although extant species are found throughout Western and Central Asia today.

#### Phelipanche

We found moderate support for a Western Asia origin of *Phelipanche* (PP = 0.41) approximately 2.8–5.5 million years ago, with alternative biogeographical hypotheses much more weakly supported (Table 1). Within the genus we found strong support for two general observations. First, important subclades of *Phelipanche* also likely originated in Western Asia or were more widespread into Europe or the Mediterranean as well. These include the large subclades designated P_1_ and P_2_ in Figure 3, the crown ancestors of which were most probably limited to Western Asia (PP = 0.38 and 0.58, respectively), but may have had a larger range extending into Europe/the Mediterranean Basin as well (PP = 0.32 and 0.34). Crown ancestors of clades nested within P2 were inferred to be limited Western Asia with even higher probability (PP > 0.7) with dispersal out of this region by some extant species (e.g., *P. gratiosa* (Webb) Carlón et al. (Canary Islands, endemic) to the Mediterranean & Europe, as well as the subclade *P. libanotica* (Schweinf. ex Boiss.) Soják + *P. reuteriana* (Rchb. fil.) Carlón et al. + *P. oxyloba* (Reut.) Soják + *P. georgii-reuteri* Carlón et al. + *P. cilicica* + *P. aegyptiaca* (Pers.) Pomel), and independent range expansions into Europe/the Mediterranean Basin from an ancestor limited to Western Asia in the sister species *P. lavandulacea* (Rchb.) Pomel and *P. mutelii* (F.W. Schultz) Pomel (PP ≥ 0.75).

Similarly, we find it most probable that the most widespread and often weedy species of *Phelipanche* had direct stem ancestors limited in range to Western Asia. These include *Phelipanche arenaria* (Borkh.) Pomel (PP = 0.71), *P. caesia* (PP = 0.5), *P. ramosa* (L.) Pomel (PP = 0.90), and *P. aegyptiaca* (PP = 0.40, with the next most probable origin as Europe/the Mediterranean, PP = 0.22).

#### Orobanche

Similar to *Phelipanche* we infer a Western Asian origin for ancestral *Orobanche* (PP = 0.43; 0.39 for *Orobanche* + *Boulardia*). Four key subclades are diagnosable by their biogeographic affinities. The first and second subclades are closely related and comprise predominantly Europe/Mediterranean species that have diversified *in situ* (O_1_ + O_2_ in Table 1, Fig. 3; ancestral range Europe/Mediterranean, PP = 0.93 and 0.99). The larger clade, also including the widespread *O. alba* and the Caucasian endemic *O. grossheimii*, likely originated in Western Asia (PP = 0.58; Fig. 3, Suppl. material 4: data S1 and Suppl. material 5: data S2) or Western Asia + Europe/the Mediterranean (PP = 0.24). A Western Asian origin is even more probable for the several more inclusive clades of *Orobanche* moving toward the root (PP = 0.66 – 0.84). The third key clade, O_3_, is composed exclusively of Caucasian endemics, except for the more widespread species *O. krylowii* (indicated as O_3_ in Fig. 3) and also originated in Western Asia (PP = 0.93, Tables 1, 2). Finally, the fourth clade (O_4_) consists of several widespread species including *O. cernua* L., *O. amoena* C.A. Mey., *O. cumana* Wallr., *O. pycnostachya* Hance, and *O. grenieri* F.W. Schultz and was also inferred to have a widespread common ancestor, though the exact geography is uncertain (Table 1). The top three most probable biogeographic states for the common ancestor encompass the regions Europe/Mediterranean plus Western Asia as well as either Central Asia, Eastern Asia, or both, but together these hypotheses only represent half of the posterior density (Table 1).

## Discussion

### Phylogenetic relationships

#### Cistanche

*C. armena* was described by Koch (1843) as *Phelypaea armena*, synonymised with *P. salsa* C.A. Mey by Boissier (1879) and transferred to *C. salsa* by Beck, where it has remained in synonym by subsequent authors (Ataei 2017; Ataei et al. 2020). However, recent morphological study has indeed shown that *C. armena* differs clearly from *C. salsa* (Piwowarczyk et al. 2019). The occurrence of this *Cistanche* species in the Caucasus requires further field and molecular studies, however at this point *C. armena* is known only from Armenia, and *C. salsa* and *C. fissa* (C.A. Mey.) Beck probably are absent from Armenia or Georgia (Piwowarczyk et al. 2019).

#### Phelypaea

This genus includes three holoparasite species (*P. coccinea*, *P. boissieri*, and *P. tournefortii*) that parasitize Asteraceae hosts. *Phelypaea coccinea*, a parasite of *Psephellus* Cass. and *Centaurea* L., rarely *Klasea* Cass., occurs in the Caucasus and Crimea, while *P. tournefortii*, a parasite of *Tanacetum* L., occurs in the Caucasus and Turkey (Piwowarczyk et al. 2019). However, *P. boissieri* shows a different distribution; it occurs in the Balkans (Albania, Greece, North Macedonia), and Western Asia (Turkey, Iraq and Iran), parasitises *Centaurea* (similarly *P. coccinea*), and occasionally *Cousinia* Cass. in Iraq (Piwowarczyk et al. 2019). The molecular (Fig. 1) and morphological features that separate *P. coccinea* and *P. boissieri* – i.e., corolla-tube short and cup-shaped; corolla-lobes broadly obovate-orbicular to orbicular, overlapping; anthers hairy (Piwowarczyk et al. 2019) – are not fully differentiating. Thus, further research into variability and the inclusion of more samples for genetic analysis are required.

#### Phelipanche

The phylogenetic relations of the newly described species, i.e., *P. zangezuri* (Piwowarczyk et al. 2018a), *P. hajastanica* (Piwowarczyk et al. 2017c), and *P. sevanensis* (Piwowarczyk et al. 2017b) are presented (Figs 1, 2).

Phylogenetic analysis of two species previously known mainly from the Mediterranean area and later found in the Caucasus, i.e., *P. portoilicitana* and *P. cernua* (Piwowarczyk et al. 2019), showed some differences between samples collected from these different parts of the range. This may indicate the ongoing process of speciation despite similarity in host association (Figs 1, 2).

ITS (Fig. 1) poorly differentiates some species aggregates in Phelipanche sect. Phelipanche, while it does well in the sect. Trionychon (Wallr.) Piwow. & Ó. Sánchez (Piwowarczyk et al. 2018d, = sect. Arenariae Teryokh.).

#### Orobanche

The recently described O. flava subsp. cicerbitae Uhlich & Rätzel [≡ *O. cicerbitae* (Uhlich et Rätzel) Tzvelev] parasitising *Cicerbita* Wallr. and *Senecio propinquus* Schischk. is distantly related to *O. flava*, at least as far as ITS (Fig. 1, Suppl. material 2: Fig. S1) and morphological (Piwowarczyk et al. 2017a) analyses have shown. On the ITS tree *O. cicerbitae* belong to clade O. subsect. Curvatae (Beck) Piwow. et al., particularly with species of the *O.* series *Krylowianae* clade (Piwowarczyk et al. 2017a). However, trees based on *trnL–trnF* sequences show displacement of samples of Caucasian *O. flava* close to *O. cicerbitae* (Fig. 2, Suppl. material 3: Fig. S2). A similar phenomenon is the placement of *trnL–trnF* sequence (Fig. 2, Suppl. material 3: Fig. S2) of *O. lycoctoni* near *O. lucorum*, a species relatively distant to *Krylowianae* species. This phenomenon may be explained by hybridisation and requires further research. In this case *O. cicerbitae* might be a species formed by the crossing of *O. flava* (or related species) as a female parent and one of the species belonging to ser. Krylowianae, but this supposition requires further study. In the Caucasus, both species often occupy the same habitats (humid tall herb vegetation) and grow with their hosts (*Caucasalia* B. Nord, *Senecio* L., *Pojarkovia* Askerova vs. *Petasites* Mill.) next to each other, thus facilitating gene flow.

We confirm that the newly described *O. javakhetica* (Piwowarczyk et al. 2018b) is distinct from the other studied species. ITS sequence does not resolve its position within other *Orobanche* species. However, trees based on *trnL–trnF* sequences (Fig. 2, Suppl. material 3: Fig. S2) show a common clade with *O. gracilis* (O. subsect. Cruentae Teryokhin) (BS = 98, PP = 1.00). Morphologically, there seems to be some similarity to the O. subsect. Orobanche (subsect. Galeatae sensu Teryokhin) and O. subsect. Curvatae (particularly with species of the O. ser. Krylowianae) (Piwowarczyk et al. 2018b). Finding other new species related to *O. javakhetica* will be helpful in confirming the phylogenetic relationships of this species.

*Orobanche schelkovnikovii* was incorrectly included in the *O.* trib./Grex *Galeatae* sensu Beck by Novopokrovskij and Tzvelev (1958). This erroneous determination of herbarium specimens collected by Schelkovnikov as *O. caryophyllacea* by Grossheim is surely the reason for its inclusion in this group. The corolla is not helmet-shaped at the apex, which clearly indicates that it should be in O. subsect. Curvatae (Piwowarczyk et al. 2019). The ITS trees indicate that *O. schelkovnikovii* belong to O. subsect. Curvatae and formed a clade with *O. centaurina* (syn. *O. kochii* F.W. Schultz, Zázvorka et al. 2019), *O. sintenisii* Beck and *O. kurdica* Boiss. & Hausskn. (syn. *O. rosea* Tzvel., Piwowarczyk et al. 2019) (Fig. 1) (BS = 99, PP = 0.98). *O. kurdica* is also morphologically most similar to *O. centaurina*, but clearly distinct based on some features and host affinity (Piwowarczyk et al. 2019). However, the sample named as *O. sintenisii* from Turkey (AY209276) was probably identified incorrectly. Based on host and locality it most likely belongs to *O. kurdica* (Fig. 1, Suppl. material 2: Fig. S1, Suppl. material 1: Table S1).

According to Novopokrovskij and Tzvelev (1958)*O. grossheimii* belongs to the group subsect. Curvatae, but the presence of dark coloured reddish or violet glandular hairs and morphology of the flowers may indicate that it belongs to the O. subsect. Glandulosae (Beck) Teryokhin (O. subsect. Glandulosae Novopokr., Piwowarczyk et al. 2019), which seems to be confirmed in our phylogenetic study (Fig. 1, Suppl. material 2: Fig. S1).

The newly described species *O. zajaciorum* (Piwowarczyk 2015) is clearly separated from other species based on ITS and *trnL–trnF* data, but its precise relationship to other *Orobanche* remains to be unclear. On the ITS tree it formed a separated clade with *O. lutea* Baumg. (subsect. Orobanche ≡ O. subsect. Galeatae sensu Teryokhin), however with low support (BS < 75) (Fig. 1). Morphologically, *O. zajaciorum* is a close relative to O. subsect. Orobanche (O. subsect. Galeatae sensu Teryokhin) – especially due to the helmet-shaped upper lip and the relatively narrowly tubular flower, however the species of *O.* Grex *Galeatae* sensu Beck (1930) are – with the exception of *O. clausonis* Pomel – much taller and have larger flowers, usually broad bidentate calyx segments, and different hosts (Piwowarczyk 2015).

*Orobanche rapum-genistae* Thuill., *O. rigens* Loisel. vs *O. colorata*/*O. anatolica* placed by Beck (1930) in Grex *Arcuatae* (O. subsect. Arcuatae Teryokhin) in our phylogenetic trees show significant discrepancy (Figs 1, 2, Suppl. material 2: Fig. S1, Suppl. material 3: Fig. S2).

*Orobanche gamosepala* is genetically very distinct, yet nested within *Orobanche*, forming a clade with *O. anatolica*/*O. colorata* (O. subsect. Arcuatae) (ITS: BS = 99, PP = 1.00, *trnL–trnF*: BS = 100, PP = 1.00) that is sister to the clade containing all other *Orobanche* species (Figs 1, 2, Suppl. material 2: Fig. S1, Suppl. material 3: Fig. S2). This species was described previously as the monotypic genus *Necranthus* Gilli from northeastern Turkey based on a calyx anatomy similar to *Boschniakia* C.A. Meyer ex Bong. and *Xylanche* Beck (Gilli 1968). Beck (1930) placed this species in trib./Grex *Galeatae*, and later Teryokhin et al. (1993) included this species in its own section O. sect. Gamosepalae Teryokh. according to its distinct calyx anatomy and seed micromorphology. In a more recent micromorphological study of Caucasian Orobanchaceae seeds, the position of *O. gamosepala* on the dendrogram is closer to *O. colorata* than to the remaining *Orobanche*, which is in accordance with the above results, and based on the shape of the seed cells, to *Cistanche* (Piwowarczyk et al. 2020b). *O. gamosepala* together with *O. colorata* and *O. anatolica* is one of the oldest lineages of Orobancheae (Table 2, see below).

According to some authors (e.g., Novopokrovskij and Tzvelev 1958; Domina and Raab-Straube 2010) *O. colorata* only grows in the countries of the Caucasus area (with ± glabrescente inflorescence), and is replaced in Turkey, Iran and Iraq by the *O. anatolica* s. str. (with ± lanate inflorescence). However, taxonomic and chorological restrictions corresponding to each taxon are not entirely clear (Piwowarczyk et al. 2019). In the Caucasus and Turkey (O. anatolica var. glabrescens Post and O. anatolica var. leucopogon (Boiss. & Hausskn. ex Boiss.) Beck) both individuals with glabrescente and lanate inflorescences are often found, and lanate indumenum is especially present when the plants are young, but not only. In the ITS trees (Fig. 1, Suppl. material 2: Fig. S1) *O. anatolica* (from Turkey) and *O. colorata* (Caucasus) formed a common clade but the difference between them remains unclear (BS = 100, PP = 1.00).

*Orobanche raddeana* is a Caucasian endemic parasitising on Campanulaceae (*Campanula* L., *Asyneuma* Griseb. & Schenk). The ITS tree may suggest that it is related to species from the subsect. Glandulosae (Fig. 1, Suppl. material 2: Fig. S1), which is also supported by morphological features, while based on *trnL–trnF* sequences (Fig. 2, Suppl. material 3: Fig. S2) *O. raddeana* forms a common clade with species mostly from the O. subsect. Curvatae ser. Krylowianae. It is worth noting that *O. raddeana*, described later as O. alba var. raddeana (Beck) Beck, is relatively distant to *O. alba*. Recently, the name *O. raddeana* was changed as a new species, *O. campanulae* Rätzel et al. (Rätzel et al. 2018), but in our opinion – despite the correction of their studies on this taxon – the traditional interpretation of the name of *O. raddeana* is valid and must be retained in order to avoid disadvantageous nomenclatural changes entailed by the strict application of the rules (Turland et al. 2018: Art. 14). For this reason, the authors of the new species (*O. campanulae*) should propose the conservation of the name *O. raddeana* according to ICN, even though other names, e.g., *O. glabrata* C.A. Mey could have priority.

Within the O. subsect. Inflatae Beck, *O. grenieri* (parasitic on mainly *Lactuca* L.) is clearly distinguished morphologically and phylogenetically from related species (*O. cernua* and *O. cumana*), as has already been shown (Piwowarczyk et al. 2015). The taxonomic relationships of the polymorphous species *O. cernua* and *O. cumana* are not entirely clear. Some researchers recognised *O. cumana* as a separate species, and others as varieties or subspecies of *O. cernua*. *O. cumana* parasitises cultivated plants, mainly *Helianthus* L. and Solanaceae (*Lycopersicon* Mill. L., *Nicotiana* L.). The problem with identifications arises when *O. cumana* parasitises wild species, i.e., *Artemisia* L. (sometimes *Xanthium* L.), like the closely allied typical *O. cernua*, because morphological differences between these two species cannot always be easily seen.

Species from subsect. Minores Teryokhin and subsect. Speciosae Teryokhin are highly polymorphic, especially regarding colour, inflorescence length and variability of flower, as well as range of hosts. In our research we used Caucasian samples of *O. laxissima* (a parasite of various tree species, i.e., *Fraxinus* L., *Carpinus* L., *Punica* L., *Robinia* L.), *O. owerinii* (a parasite of herbaceous hosts, i.e., *Trifolium* L., *Vicia* L.), and *O. minor* (samples from *Chondrilla* L. and *Lactuca* hosts) (Figs 1, 2). Tzvelev considered that *O. owerinii* is a polymorphic species, represented by several races, which can be treated as distinct species. *O. owerinii* is probably closely related to *O. crenata* Forssk. and replaces it in the upper montane zone of Asia Minor and Caucasian territories (Novopokrovskij and Tzvelev 1958). However, the typical *O. crenata* has not been confirmed in the Caucasus. *O. laxissima* seems to be very similar morphologically to *O. transcaucasica* Tzvel., apparently also a parasite on shrubs and trees and described in a hornbeam-oak forest from the Shemackha region in Azerbaijan by Tzvelev (1957). Rätzel and Uhlich (2004) mistakenly assigned *O. laxissima* to the O. subsect. Galeatae sensu Teryokhin (O. trib. Galeatae sensu Beck). Morphologically (Piwowarczyk et al. 2019) and molecularly (Figs 1, 2, Suppl. material 2: Fig. S1, Suppl. material 3: Fig. S2), the species clearly belongs to the O. subsect. Speciosae (*O.* trib./Grex *Speciosae* sensu Beck). *O. laxissima* can be confused by an inexperienced researcher with *O. owerinii*, especially in the herbarium materials without details about the host. Our results support this affinity (Figs 1, 2, Suppl. material 2: Fig. S1, Suppl. material 3: Fig. S2). Moreover, *O. laxissima*, *O. owerinii* seem to be phylogenetically similar, especially on ITS trees, with several other species. The recent diversification of these lineages (Tables 1, 2, Fig. 3) could explain why some of these species have not entirely diverged.

Molecular studies do not indicate the validity of dividing species into subsect. Speciosae because the species included here are both very morphologically and genetically similar to the subsect. Minores. Similar conclusions can be used to merit the inclusion of *Vitellinae* Teryokhin, *Hederae* Teryokhin, and *Camptolepides* Teryokhin in separate subsections when they are clearly similar to species from the subsect. Minores and *Inflatae* (respectively). The results presented here suggest that the currently distinguished systematic division of *Orobanche*-based morphology is frequently inconsistent with the phylogenetic studies and thus needs revision, regarding both phenotypic traits and molecular analyses, for example, the heterogeneous subsect. Curvatae is clearly resolved as polyphyletic (Fig. 1). Furthermore, our knowledge of some poorly understood species in sections in the *Phelipanche* genus requires further taxonomic, field (especially in Western and Central Asia), and molecular research.

### Biogeography

We found strong support for Western Asia as the centre of origin for large subclades of *Phelipanche*, *Orobanche*, and *Cistanche* (Table 1, Fig. 3), followed by both diversification *in situ* as well as dispersal out of this region over the last 1–2 million years (Table 2, Fig. 3). This supports previous hypotheses that Western Asia, especially the Caucasus region and nearby high mountains in the Middle East and Western Asia, as important centres of origin for Eurasian Orobancheae (Rätzel and Uhlich 2004), despite our uncertainty in the biogeography of the ultimate common ancestor of *Orobanche* + *Phelipanche + Aphyllon*. About 30 species of holoparasitic Orobancheae are known as endemic (15 confirmed species) to the Caucasus or have most of their range there. High-mountain genetic lineages with subalpine habitats are especially unique, such as species from the ser. Krylowianae (*O. inulae*, *O. mlokosiewiczii*, *O. cicerbitae*, and *O. arpica*), as well as *O. gamosepala*, *O. grossheimii*, *O. raddeana*, *O. javakhetica*, *O. schelkovnikovii*, and *O. zajaciorum*. The extant diversity in this region is a combination of clades of recently diverged (i.e., neoendemic) species such as those in ser. Krylowianae, as well as species on relatively long branches without close relatives (e.g., *O. raddeana*, *O. javakhetica* and *Phelipanche bungeana* (Beck) Soják) or species-poor clades subtended by long branches (e.g., *Phelypaea*, or *O. anatolica* + *O. colorata*; Table 2). Thus, the Caucasus region may be considered as one of mixed endemism for parasitic Orobancheae (sensu Mishler et al. 2014). However, we do not have the temporal resolution to determine if these long-branch parasite species have always been range-limited, or have gone through expansions and subsequent contraction due to climatic or other ecological shifts.

The broader floristic and geological history of the Caucasus and high mountain region does provide some clues to the processes that its status as a centre of extant diversity, a centre of origin for large portions of this diversity, and potentially a region of mixed endemism for holoparasitic Orobancheae. The Caucasus has an unusually high proportion of endemic and relict species for a continental, non-tropical region (Tarkhnishvili 2014). Approximately 25% of vascular plant species found in the Caucasus are endemic, as well as unique vegetation types such as Colchic and Hyrcanian forests with relict tree species (Kikvidze and Oshawa 2001). The Caucasian oreoxerophytic flora has a historical connection with the Mediterranean and Asia Minor due to Pleistocene migration from Asia Minor eastwards. Following the retreat of the glaciers, xerophytic flora from the Irano-Turanian region and mountains of Central Asia also migrated to the Caucasus, with simultaneous degradation of the mesothermophilous forest vegetation (Nakhutsrishvili and Abdaladze 2005). The southern part of the Caucasus in Armenia is also located in the Irano-Anatolian biodiversity hotspot (as well as northeastern Iran and Iraq, and central and eastern Turkey). This is the only global biodiversity hotspot entirely inside Southwest Asia (Noroozi et al. 2018), with over 40% endemic plant species (Mittermeier et al. 2005). Longstanding explanations for the unique flora in this region highlight the role the Caucasus and high mountains have played as a refugium for many elements of the pre-glacial Tertiaty flora during cooling of the Pliocene and Pleistocene, and aridification during the Upper Pleistocene and Holocene (Kuznetsov 1909; Fedorov 1952; Kharadze 1960). Along with aridification, another important contributor to the flora was the Pleistocene migration of plants from Asia Minor and post-glacial xerophytic migrants from the mountains of central Asia (Nakhutsrishvili and Abdaladze 2005; Zernov 2006). Characteristics such as a dissected, heterogeneous topography, a large altitudinal range, and a relatively mild climate subsequently helped preserve these floristic elements (Kikvidze and Oshawa 2001). Consistent with this pattern, some of the oldest lineages of Orobancheae that include extant Caucasus endemics were probably also found in Western Asia at their time of divergence during the Pliocene and Pleistocene (Tables 1, 2, Fig. 3). These refugia may also explain the disjunct ranges of many *Orobanche* and *Phelipanche* species present both in Western Asia (especially the Caucasus) and further west in Europe, such as *O. grenieri*, *P. cernua*, *P. portoilicitana* and the Carpathian mountain species *O. flava* (Piwowarczyk et al. 2019).

However, we also found a number of very recent diversification events in Orobancheae, pointing to recent *in situ* speciation as a complementary mechanism that explains the high levels of endemism in this region (Table 2, Fig. 3). For example, the diversification of the *Orobanche laxissima* + *O. owerinii* + *O. transcaucasica* clade was exceptionally recent (most likely in the last 150,000 years, but this may exceed the precision of our analysis). Many species of Orobanchaceae are associated with calcareous habitats that probably favour speciation and are abundant throughout the Caucasus (Kikvidze and Oshawa 2001). Many of the same geographical and ecological factors that have made western Asia a refugium for *Orobanche* and *Phelipanche* species also likely contributed to subsequent diversification, such as topographic and habitat heterogeneity, and a diverse flora of potential host species, many of which are also endemic or of limited range. In the postglacial, continental climate of western Asia, suitable xerophytic habitat has replaced forest in many areas. The expansion of steppe, subalpine, subalpine or steppe communities – and more importantly the potential host plants occupying them – may explain the diversification of Orobancheae in this region.

By contrast, the biodiversity of *Orobanche* and *Cistanche* that evolved in Europe and especially in the Mediterranean Basin appears to have done so more recently than that in West Asia, although we cannot confidently infer ancestral states of lineages greater than 5 million years (Table 1, Fig. 3). Iberia, Italy, and the Balkans are three well-studied refugial regions of Mediterranean Europe where thermophilic species persisted through glacial periods, (Bennett et al. 1991; Comes and Kadereit 1998, 2003; Taberlet et al. 1998; Hewitt 1999; Habel et al. 2014) however our analysis is not fine-grained enough to distinguish among them. Collectively, the Mediterranean region has been recognised as another of the world hotspots of biodiversity, with more than 25,000 known vascular plant species, mostly endemic. The Mediterranean flora consists of low species-genus ratios, with many primarily long‐lived taxa restricted to island or mountain habitats, probably as paleoendemics of likely Tertiary origin (Blondel and Aronson 1999). This unique plant diversity and endemism are the result of several key factors: paleogeological and climatic history, biogeography, and ecogeographical heterogeneity, with evidence that adaptive radiation has taken place relatively recently (Blondel and Aronson 1999; Comes 2004). Several radiations within *Orobanche* and *Cistanche* have taken place over the last million years (Table 1, Fig. 3), but the clade of *O. foetida* Poir., *O. densiflora* Salzm. ex Bertol., *O. sanguinea* C. Presl, *O. austrohispanica* M.J.Y. Foley, and the more widespread *O. gracilis* likely began diversifying earlier, about 2–3 million years ago. Like the origin of many other Mediterranean flora elements, the ancestors of each of these clades came from western Asia, then moved into the Mediterranean and diversified (Fig. 3; Blondel and Aronson 1999). An alternative hypothesis, more strongly supported for *Phelipanche* than *Orobanche*, is that a widespread ancestor growing across Europe and western Asia may have given rise to both Western Asian and European/Mediterranean clades (the second most probable ancestral states of many *Phelipanche* and *Orobanche* common ancestors, Suppl. material 4: data S1 and Suppl. material 5: data S2).

We conclude with a cautionary note that we were not able to exhaustively sample the Orobancheae, in particular certain species of *Orobanche*, such as O. sect. Kotschyinae Teryokhin from the Middle East and western and central Asia. The addition of certain other lineages, such as species in O. subsect. Coerulescentes Teryokhin would likely strengthen the importance of diversification in East Asia. Finally, our results within *Cistanche* are sensitive to changing taxonomic concepts.

## Supplementary materials

Supplementary material 1 Table S1 Data type List of taxa and sequences analysed Explanation note List of taxa and sequences analysed (* sequences obtained from GenBank). https://binary.pensoft.net/file/519658This dataset is made available under the Open Database License (http://opendatacommons.org/licenses/odbl/1.0/). The Open Database License (ODbL) is a license agreement intended to allow users to freely share, modify, and use this Dataset while maintaining this same freedom for others, provided that the original source and author(s) are credited.Renata Piwowarczyk, Adam C. Schneider, Grzegorz Góralski, Dagmara Kwolek, Magdalena Denysenko-Bennett, Anna Burda, Karolina Ruraż, Andrzej J. Joachimiak, Óscar Sánchez Pedraja

Supplementary material 2 Figure S1 Data type phylogenetic tree Explanation note Rooted Bayesian phylogenetic consensus tree inferred from ITS sequences (outgroup: *Lindenbergia sinaica*). Numbers near branches show Bayesian posterior probabilities ≥ 0.75. The bar represents the amount of genetic change (nucleotide substitutions per site). Species names, the country of origin, host species (if available) and GenBank accession number are included on the phylogeny tip labels. https://binary.pensoft.net/file/519659This dataset is made available under the Open Database License (http://opendatacommons.org/licenses/odbl/1.0/). The Open Database License (ODbL) is a license agreement intended to allow users to freely share, modify, and use this Dataset while maintaining this same freedom for others, provided that the original source and author(s) are credited.Renata Piwowarczyk, Adam C. Schneider, Grzegorz Góralski, Dagmara Kwolek, Magdalena Denysenko-Bennett, Anna Burda, Karolina Ruraż, Andrzej J. Joachimiak, Óscar Sánchez Pedraja

Supplementary material 3 Figure S2 Data type phylogenetic tree Explanation note Rooted Bayesian phylogenetic consensus tree constructed using plastid *trnL–trnF* spacer sequences (outgroup: *Lindenbergia sinaica*). Numbers near branches show Bayesian posterior probabilities ≥ 0.75. The bar represents the amount of genetic change (nucleotide substitutions per site). Species names, the country of origin, host species (if available) and GenBank accession number are included on the phylogeny tip labels. https://binary.pensoft.net/file/519660This dataset is made available under the Open Database License (http://opendatacommons.org/licenses/odbl/1.0/). The Open Database License (ODbL) is a license agreement intended to allow users to freely share, modify, and use this Dataset while maintaining this same freedom for others, provided that the original source and author(s) are credited.Renata Piwowarczyk, Adam C. Schneider, Grzegorz Góralski, Dagmara Kwolek, Magdalena Denysenko-Bennett, Anna Burda, Karolina Ruraż, Andrzej J. Joachimiak, Óscar Sánchez Pedraja

Supplementary material 4 Data S1 Data type DNA sequence alignment (fasta format) Explanation note Sequence matrix used for biogeography analysis. https://binary.pensoft.net/file/519661This dataset is made available under the Open Database License (http://opendatacommons.org/licenses/odbl/1.0/). The Open Database License (ODbL) is a license agreement intended to allow users to freely share, modify, and use this Dataset while maintaining this same freedom for others, provided that the original source and author(s) are credited.Renata Piwowarczyk, Adam C. Schneider, Grzegorz Góralski, Dagmara Kwolek, Magdalena Denysenko-Bennett, Anna Burda, Karolina Ruraż, Andrzej J. Joachimiak, Óscar Sánchez Pedraja

Supplementary material 5 Data S2 Data type tree file Explanation note Historical biogeography reconstruction (maximum clade credibility tree). https://binary.pensoft.net/file/519662This dataset is made available under the Open Database License (http://opendatacommons.org/licenses/odbl/1.0/). The Open Database License (ODbL) is a license agreement intended to allow users to freely share, modify, and use this Dataset while maintaining this same freedom for others, provided that the original source and author(s) are credited.Renata Piwowarczyk, Adam C. Schneider, Grzegorz Góralski, Dagmara Kwolek, Magdalena Denysenko-Bennett, Anna Burda, Karolina Ruraż, Andrzej J. Joachimiak, Óscar Sánchez Pedraja
