# Supplementary material for: Phylogeny and historical biogeography analysis support Caucasian and Mediterranean centres of origin of key holoparasitic Orobancheae (Orobanchaceae) lineages
Source: PhytoKeys. 2021 Mar 12;174:165–94. doi: 10.3897/phytokeys.174.62524 (PMC7979677; doi:10.3897/phytokeys.174.62524)
Supplement: Supplementary material 1 — Table S1 [file phytokeys-174-165-s001.pdf]

Taxon	ITS	*trnL-trnF*	PhyA	PhyB	Country	Voucher information or reference	Host
Aphyllon							
A. arizonicum	KX161146*	KX161298*			USA	Schneider et al. 2016	
A. californicum subsp. *californicum*	KX161153*	KX161306*			USA	Schneider et al. 2016	*Grindelia stricta*
A. californicum subsp. *californicum*			AM233989*		USA	Bennett and Mathews 2006	
A. californicum subsp. *californicum*				KC542244*	USA	McNeal et al. 2013	
A. californicum subsp. *condensum*	KX161160*	KX161307*			USA	Schneider et al. 2016	*Heterotheca villosa* var. *minor*
A. californicum subsp. *feudgei*	KX161163*	KX161308*			USA	Schneider et al. 2016	
A. californicum subsp. *grande*	KX161170*	KX161309*			USA	Schneider et al. 2016	*Lessingia filaginifolia*
A. californicum subsp. *grayanum*	KX161172*	KX161310*			USA	Schneider et al. 2016	*Eucephalus breweri*
A. californicum subsp. *jepsonii*	KX161176*	KX161311*			USA	Schneider et al. 2016	*Artemisia tridentata*
A. chilense	EU600371*				Argentina	Park et al. 2008	
A. chilense		KX161314*			Argentina	Schneider et al. 2016	*Grindelia ventanensis*
A. cooperi subsp. *cooperi*	KX161186*	KX161317*			Mexico	Schneider et al. 2016	*Encelia farinosa*
A. cooperi subsp. *palmeri*	KX161187*				USA	Schneider et al. 2016	*Viguiera stenoloba*
A. corymbosum	KX161191*	KX161320*			USA	Schneider et al. 2016	*Artemisia*
A. corymbosum			AM233990*		USA	Bennett and Mathews 2006	
A. corymbosum				KC542245*	USA	McNeal et al. 2013	
A. dugesii	KX161195*	KX161322*			Mexico	Schneider et al. 2016	
A. epigalium subsp. *epigalium*	KX161211*	KX161375*			USA		*Galium*
A. epigalium subsp. *notocalifornicum*	KX161207*	KX161373*			USA	Schneider et al. 2016	*Galium andrewsii*
A. fasciculatum	KX161208*	KX161324*			USA	Schneider et al. 2016	*Artemisia frigida*
A. fasciculatum			AM233992*		USA	Bennett and Mathews 2006	

A. fasciculatum				KC542248*	USA	McNeal et al. 2013	
A. franciscanum	KX161220*	KX161332*			USA	Schneider et al. 2016	
A. ludovicianum	KX161227*	KX161312*			USA	Schneider et al. 2016	*Ambrosia*
A. ludovicianum			KC542191*	KC542246*	USA	McNeal et al. 2013	
A. multiflorum	KX161230*	KX161336*			USA	Schneider et al. 2016	*Heterotheca latifolia*
A. parishii subsp. *brachylobum*	KX161242*	KX161339*			USA	Schneider et al. 2016	
A. parishii subsp. *parishii*	KX161244*	KX161341*			USA	Schneider et al. 2016	*Gutierrezia microcephala*
A. pinorum	AY209292*				USA	Schneeweiss et al. 2004	
A. pinorum			AM233994*		USA	Bennett and Mathews 2006	
A. pinorum				KC542247*	USA	McNeal et al. 2013	
A. purpureum	KX161266*	KX161348*			USA	Schneider et al. 2016	*Sedum obtusatum*
A. riparium	KX161253*	KX161343*			USA	Schneider et al. 2016	*Xanthium strumarium* and *Ambrosia artemisiifolia*
A. robbinsii	KX161262*	KX161378*			USA	Schneider et al. 2016	*Eriophyllum staechadifolium*
A. sp. on *Zaluzania*	KX161229*	KX161335*			Mexico	Schneider et al. 2016	*Zaluzania triloba*
A. tacnaense	KX161263*	KX161345*			Peru	Schneider et al. 2016	
A. tarapacanum	KX161264*	KX161346*			Chile	Schneider et al. 2016	
A. tuberosum	KX161148*	KX161300*			USA	Schneider et al. 2016	*Adenostoma fasciculatum*
A. uniflorum	KX161280*	KX161361*			USA	Schneider et al. 2016	*Solidago canadensis*
A. validum subsp. *howellii*	KX161291*	KX161364*			USA	Schneider et al. 2016	
A. validum subsp. *validum*	KX161292*	KX161367*			USA	Schneider et al. 2016	
A. vallicola	KX161295*	KX161369*			USA	Schneider et al. 2016	*Sambucus mexicana*
Boschniakia							

B. himalaica	AY911212*					Wolfe et al. 2005	
B. himalaica			KC542187*	KC542204*		McNeal et al. 2013	
B. rossica	AY911214*					Wolfe et al. 2005	
B. rossica			AM233932*			Bennett and Mathews 2006	
B. rossica				KC542206*		McNeal et al. 2013	
Boulardia							
B. latisquama (O. macrolepis)	AY209286*				Spain	Schneeweiss et al. 2004	*Rosmarinus*
B. latisquama (O. macrolepis)	AY209288*				Spain	Schneeweiss et al. 2004	*Rosmarinus*
Cistanche							
C. aff. lutea 2 (C. algeriensis ined.)	LT715434*	LT714889*			Algeria	Ataei 2017	
C. aff. lutea 1 (= C. almeriensis ined.)	LT715432*	LT714887*			Egypt	Ataei 2017	
C. ambigua	LT715397*	LT714849*			Afghanistan	Ataei 2017	
C. armena	KY062761*	MT027259			Armenia	Piwowarczyk et al. 2017d/ Khor Virap, 26 May 2016, R. Piwowarczyk (KTC)	*Alhagi maurorum*
C. armena	MT026622				Armenia	Khor Virap, 26 May 2016, *R. Piwowarczyk* (KTC)	*Salsola dendroides*
C. aff. fissa 1 (= *C. bamianica ined.*)	LT715388*	LT714841*			Afghanistan	Ataei 2017	
C. aff. fissa 2 (= C. bilobata ined.)*		LT714840*			Azerbaijan	Ataei 2017	
C. brunneri	LT715431*	LT714908*			Senegal	Ataei 2017	
C. aff. tubulosa (= C. chabaharensis ined.)*	LT715487*	LT714943*			Yemen	Ataei 2017	
C. deserticola	LT715387*	LT714839*			Kyrgyzstan	Ataei 2017	
C. deserticola	KY706617*					Fu et al. 2017	
C. flava subsp. nov. (=*Cistanche flava* subsp. *brevibracta ined.)*	LT715534*	LT714994*			Iran	Ataei 2017	

C. flava subsp. *flava*	LT715552*	LT715013*			Iran	Ataei 2017	
C. laxiflora subsp. nov (*C. laxiflora* subsp. *alba*	LT715464*	LT714920*			Iran	Ataei 2017	
C. laxiflora subsp. *laxiflora*	LT715469*	LT714925*			Iran	Ataei 2017	
C. lutea	AY209302*				Spain	Schneeweiss et al. 2004	
*C. mauritanica (*incl. *C. macrocalycinum)*	LT715410*	LT714862*			Algeria	Ataei 2017	
C. aff. ridgewayana 1 (= C. persica ined.)*	LT715400*	LT714852*			Iran	Ataei 2017	
C. phelypaea subsp. nov. (*C. phelypaea* subsp. *naviculata ined.)*	LT715415*	LT714868*			Morocco	Ataei 2017	
C. phelypaea subsp. *phelypaea*	LT715417*	LT714870*			Morocco	Ataei 2017	
C. phelypaea subsp. *phelypaea*	AY209303*				Spain	Schneeweiss et al. 2004	
C. ridgewayana	LT715399*	LT714851*			Afghanistan	Ataei 2017	
C. rosea	LT715460*	LT714916*			South Arabia	Ataei 2017	
C. armena (= C. salsa sensu Ataei)	LT715385*	LT714837*			Armenia	Ataei 2017	
C. salsa	AB217872*				China	Unpublished	
C. senegalensis	LT715481*	LT714937*			Tanzania	Ataei 2017	
C. sinensis	LT715381*	LT714833*			Mongolia	Ataei 2017	
C. sinensis	KY706618*				China	Fu et al. 2017	
C. lutea (= C. tinctoria ined.)*	LT715455*	LT714911*			Algeria	Ataei 2017	
C. lutea (= C. tinctoria ined.)*	KF850607*				Saudi Arabia	Unpublished	
C. aff. ridgewayana 1 (C. tomentosa subsp. *longibracta)*	LT715403*	LT714855*			Iran	Ataei 2017	
C. aff. ridgewayana 2 (C. tomentosa subsp. *longibracta)*	LT715404*	LT714856*			Iran	Ataei 2017	
C. tubulosa subsp. nov . *(=C. tubulosa subsp. iranica ined.*)*	LT715490*	LT714946*			Iran	Ataei 2017	

C. tubulosa subsp. *tubulosa*	LT715529*	LT714989*			Iran	Ataei 2017	
C. violacea	LT715429*	LT714883*			Tunisia	Ataei 2017	
C. violacea	AY209305*				Morocco	Schneeweiss et al. 2004	
Conopholis							
C. alpina	KC480324*			KC542216*	USA	McNeal et al. 2013	
C. alpina			AM233949*		USA	Bennett and Mathews 2006	
C. americana	AY209289*				USA	Schneeweiss et al. 2004	
C. americana			AM233950*		USA	Bennett and Mathews 2006	
C. americana				KC542217*	USA	McNeal et al. 2013	
Epifagus							
E. virginiana	AY209290*				USA	Schneeweiss et al. 2004	
E. virginiana			AM233954*		USA	Bennett and Mathews 2006	
E. virginiana				KC542221*	USA	McNeal et al. 2013	
Gleadovia							
G. mupinensis	KY706614*		KY706629*	KY706631*	China	Fu et al. 2017	
Kopsiopsis							
K. hookeri	AY209291*				USA	Schneeweiss et al. 2004	
K. hookeri			AM233931*			Bennett and Mathews 2006	
K. hookeri				KC542205*		McNeal et al. 2013	
K. strobilacea	AY911215*					Wolfe et al. 2005	
K. strobilacea			AM233933*			Bennett and Mathews 2006	
K. strobilacea				KC542207*		McNeal et al. 2013	

Lindenbergia							
L. sinaica	KY513938*	KX524675*			Israel	Piwowarczyk et al. 2018d / Kwolek et. al 2017	
Mannagettaea							
M. hummelii	KC480355*					McNeal et al. 2013	
Orobanche							
O. alba	MT026639	MT027260			Georgia	Sighnaghi, 28 May 2014, *R. Piwowarczyk* (KTC)	*Origanum vulgare*
O. alba	MT026631	MT027261			Georgia	Tbilisi, 25 May 2014, *R. Piwowarczyk* (KTC)	*Thymus*
O. alba	KY218658*	MT027262			Armenia	Piwowarczyk et al. 2017a / Aragats Mt., 8 July 2015, *R. Piwowarczyk* (KTC)	*Thymus*
O. alba	KY513961*	KU238873*			Poland	Piwowarczyk et.al. 2018d / Kwolek et al. 2017	*Thymus pulegioides*
O. alba	KY513962*	KY484495*			Poland	Piwowarczyk et al. 2018d	*Salvia verticillata*
O. alsatica	KY513937*	KY484482*			Poland	Piwowarczyk et al. 2018d	*Peucedanum cervaria*
O. amethystea	AY209274*				Spain	Schneeweiss et al. 2004	*Eryngium campestre*
O. amoena	MT321794				Kazakhstan	*Rjeznitschenko* (LE)	
O. anatolica (O. colorata)	AY209282*				Turkey	Schneeweiss et al. 2004	*Salvia*
O. anatolica (O. colorata)	AY209283*				Turkey	Schneeweiss et al. 2004	*Salvia*
O. arpica	MH100853*	MH100977*			Armenia	Piwowarczyk et al. 2018c	*Psephellus pulcherrimus*
O. arpica	MH100854*	MH100978*			Armenia	Piwowarczyk et al. 2018c	*Psephellus pulcherrimus*
O. artemisiae-campestris	KR260921*	KY484484*			Czech Republic	Piwowarczyk et al. 2018d	*Artemisia campestris*

O. austrohispanica	AY209241*				Spain	Schneeweiss et al. 2004	
O. ballotae	AY960729*				Spain	Carlón et al. 2005	*Ballota hirsuta*
O. bartlingii	MT026637	MT027283			Georgia	N of Udabno, 26 May 2014, *R. Piwowarczyk* (KTC)	*Bilacunaria*
O. bartlingii	KY218659*	MT027284			Georgia	Piwowarczyk et al. 2017a / Abastumani, 12 July 2015, *R. Piwowarczyk* (KTC)	*Seseli libanotis*
O. bartlingii	KY513933*	KY484478*			Poland	Piwowarczyk et al. 2018d	*Seseli libanotis*
O. boninsimae (Platypholis boninsimae)	KU647698*		KU647700*	KU647701*	Japan	Li et al. 2017	
O. caryophyllacea	KY513958*	KY484465*			Poland	Piwowarczyk et al. 2018d	*Galium odoratum*
O. caryophyllacea	KY513941*	KU238869*			Poland	Piwowarczyk et al. 2018d / Kwolek et al. 2017	*Galium boreale*
O. caryophyllacea	MT026653	MT027317			Armenia	Amberd, Aragats Mt, 8 July 2015, *R. Piwowarczyk* (KTC)	*Galium verum*
O. caryophyllacea	MT026656	MT027318			Armenia	Antarut, Aragats Mt, 8 July 2015, *R. Piwowarczyk* (KTC)	*Galium verum*
O. centaurina (O. ritro)		KY765938*			Russia	Piwowarczyk et al. 2018d	*Echinops ritro*
O. centaurina (O. kochii)	KY513939*	KY484469*			Poland	Piwowarczyk et al. 2018d	*Centaurea scabiosa*
O. centaurina (O. kochii)	KY513942*	KY484471*			Poland	Piwowarczyk et al. 2018d	*Centaurea scabiosa*
O. centaurina (O. kochii)	MT026609	MT027287			Russia	Crimea, Echkidag Mts, Delyamet-Kaya, 24 June 2019, *A.V. Fateryga, S.A. Svirin, P.E. Yevseyenkov* (MW)	*Ptilostemon echinocephalus*
O. cernua var. *australiana*	AY209230*				Australia	Carlón et al. 2005	
O. cernua var. *desertorum*	AY960731*				Algieria	Carlón et al. 2005	
O. cernua var. *cumana*	KP641332*	KU238874*			Georgia	Piwowarczyk et al. 2015 / Kwolek et al. 2017	*Artemisia*
O. cernua var. *cumana*	AY209231*				Georgia	Schneeweiss et al. 2004	*Artemisia meyeriana*
O. cernua var. *cumana*	KP641333*	MT027325			Georgia	Piwowarczyk et al. 2015 / between Rustavi and Idumala, 3 June 2014, *R. Piwowarczyk* (KTC)	*Artemisia*
O. cernua var. *cumana (O. cumana var. helianthi)*	MK024277*				Bulgaria	Unpublished	*Helianthus*
O. cernua var. *cumana*	KC811226*				China	Unpublished	*Lycopersicon*
O. cernua var. *cumana*	AY209231*				Georgia	Schneeweiss et al. 2004	*Artemisia mayeriana*

O. cernua var. *cernua*	AY960726*				Spain	Carlón et al. 2005	
O. cernua var. *cernua*	EU655626*				Israel	Carlón et al. 2008	
O. cicerbitae	MT026598	MT027307			Georgia	near Shdugra waterfall, N of Mazeri, 25 July 2018, *R. Piwowarczyk* (KTC)	*Pojarkovia pojarkovae*
O. cicerbitae	MT026600	MT027309			Georgia	N of Mazeri, 25 July 2018, *R. Piwowarczyk* (KTC)	*Caucasalia*
O. cicerbitae	MT026590	MT027315			Georgia	Mestia, in direction to Koruldi lake trial, 22 July 2018, *R. Piwowarczyk* (KTC)	*Pojarkovia pojarkovae*
O. cicerbitae	MT026599	MT027308			Georgia	N of Mazeri, in direction to Ushba Mt., 25 July 2018, *R. Piwowarczyk* (KTC)	*Pojarkovia pojarkovae*
O. cicerbitae	MT026605	MT027314			Russia	12 July 2001, *S. Rätzel* (B)	*Cicerbita*
O. cicerbitae	MT026606	MT027316			Azerbaijan	11 June 2013, *S. Rätzel* (B)	*Senecio*
O. clausonis	MT026594	MT027319			Spain	Gaucín, 2014, *G. Gómez Cesares & G. Moreno Moral* (herb. Ó. Sánchez Pedraja)	*Galium*
O. coerulescens	KY513935*	KU238865*			Poland	Piwowarczyk et al. 2018d / Kwolek et al. 2017	*Artemisia campestris*
O. coerulescens	KY218643*	KU238864*			Poland	Piwowarczyk et al. 2017a / Kwolek et al. 2017	*Artemisia campestris*
O. coerulescens	MG979752*	KU238875*			Georgia	Piwowarczyk et al. 2018b / Kwolek et al. 2017	*Artemisia*
O. coerulescens	MT026655	MT027326			Armenia	between Semyonovka and Tsovagyugh, 9 July 2015, *R. Piwowarczyk* (KTC)	*Artemisia*
O. colorata	KY218653*	MG987210*			Georgia	Piwowarczyk et al. 2017a / Piwowarczyk et al. 2018b	*Salvia verticillata*
O. colorata	MT026595	MT027329			Armenia	S of Kajaran, 3 June 2016, *R. Piwowarczyk* (ERCB, KTC)	*Salvia verticillata*
O. colorata	AY209281*				Georgia	Schneeweiss et al. 2004	*Salvia*
O. crenata	AY209266*				Greece	Schneeweiss et al. 2004	
O. crenata	EU655607*				Israel	Carlón et al. 2008	
O. crenata	DQ458909*				Spain	Unpublished	*Lactuca sativa*
O. densiflora	AY960725*				Spain	Carlón et al. 2005	*Lotus creticus*
O. densiflora	AY209243*				Spain	Schneeweiss et al. 2004	*Lotus creticus*
O. densiflora			AM233991*			Bennett and Mathews 2006	

O. ebuli	MT026588	MT027263			Italy	Latium region, Tancia Mt., 2015, *H. Uhlich*	*Sambucus ebulus*
O. elatior	KY218644*	KU328866*			Poland	Piwowarczyk et al. 2017a / Kwolek et al. 2017	*Centaurea scabiosa*
O. elatior	KY513969*	KY484474*			Poland	Piwowarczyk et al. 2018d	*Centaurea scabiosa*
O. flava	KY218652*	KY484492*			Poland	Piwowarczyk et al. 2017a / Piwowarczyk et al. 2018d	*Petasites kablikianus*
O. flava	KY513959*	KY484494*			Poland	Piwowarczyk et al. 2018d	*Petasites kablikianus*
O. flava	KY513955*	KY484493*			Slovakia	Piwowarczyk et al. 2018d	*Petasites kablikianus*
O. flava	MT026602	MT027311			Georgia	Shovi, near the trail to Udziro lake, 18 July 2018, *R. Piwowarczyk* (KTC)	*Petasites, Caucasalia*
O. flava	MT026603				Georgia	Shovi, near the trail to Udziro lake, 18 July 2018, *R. Piwowarczyk* (KTC)	*Petasites*
O. flava	AY209255*				Georgia	Schneeweiss et al. 2004	cf. *Petasites (Tussilago farfara)*
O. flava	MT026608	MT027312			Russia	Dagestan, Buynaksk distr., Manasaul, 24 June 2018, *A.V. Fateryga* (MW)	*Petasites*
O. foetida	EU655603*				Spain	Unpublished	*Erophaca*
O. gamosepala	MT026589	MT027330			Armenia	Akhundov [Pyunik] – Takarlu [Artavaz], 15 June 1965, *Z. Gevorkyan* (ERCB)	*Geranium*
O. gamosepala	MT026604	MT027331			Georgia	Shovi, 18 July 2018, *R. Piwowarczyk* (KTC)	*Geranium*
O. gracilis	KY218648*	KU238871*			Austria	Piwowarczyk et al. 2017a / Kwolek et al. 2017	*Anthyllis vulneraria, Dorycnium germanicum*
O. gracilis	JX193303*				Austria	Piednoël et al. 2012	*Chamaecytisus* sp.
O. gracilis	AY209238*				Spain	Schneeweiss et al. 2004	
O. gracilis		NC_023464*				Wicke et al. 2013	*Chamaecytisus* sp.
O. gracilis	MT026640	MT027327			Georgia	Kortaneti, 31 May 2014, *R. Piwowarczyk* (KTC)	*Cytisus*
O. gracilis			AM233993*			Bennett and Mathews 2006	
O. grenieri	KP641335*	KU238876*			Georgia	Piwowarczyk et al. 2015 / Kwolek et al. 2017	*Lactuca*
O. grenieri	KP641334*				Spain	Piwowarczyk et al. 2015	*Lactuca*
O. grossheimii	AY209277*				Georgia	Schneeweiss et al. 2004	*Cephalaria gigantea*

O. grossheimii	MT026632	MT027320			Georgia	N of Modega, 1 June 2014, *R. Piwowarczyk* (KTC)	*Cephalaria gigantea*
O. grossheimii	MT026596	MT027321			Armenia	Ghazanchi, 22 July 2017, *R. Piwowarczyk* (KTC)	*Cephalaria gigantea*
O. haenseleri	AY209253*				Spain	Schneeweiss et al. 2004	*Helleborus foetidus*
O. hederae	KY513931*	KY484472*			Montenegro	Piwowarczyk et al. 2018d	*Hedera helix*
O. hederae	AY209273*				Georgia	Schneeweiss et al. 2004	*Hedera*
O. inulae	MT113106	MT118663			Georgia	Shovi, 18 July 2018, *R. Piwowarczyk* (KTC)	*Inula*
O. inulae	MH100855*	MH100979*			Georgia	Piwowarczyk et al. 2018c	*Inula*
O. javakhetica	MG979750*	MG987214*			Armenia	Piwowarczyk et al. 2018b	*Lomelosia caucasica; Stachys macrantha*
O. javakhetica	MG979751*	MG987209*			Armenia	Piwowarczyk et al. 2018b	*Lomelosia caucasica; Stachys macrantha*
O. javakhetica	MT026601	MT027328			Georgia	Ushguli, 24 July 2018, *R. Piwowarczyk* (KTC)	*Stachys macrantha*
O. krylowii	KF359500*				Albany	Frajman et al. 2013	*Thalictrum minus*
O. krylowii	KY218645*				Russia	Piwowarczyk et al. 2017a	*Thalictrum*
O. krylowii	KF359501*				Russia	Frajman et al. 2013	
O. krylowii	KY218661*	MG987212			Russia	Piwowarczyk et al. 2017a / Irkutsk district, Zaliv Yershovskiy, 16 Aug 1990, *M.M. Ivanova* (IRKU)	*Thalictrum*
O. kurdica	MT026641	MT027285			Armenia	Amberd, Aragats Mt, 8 July 2015, *R. Piwowarczyk* (KTC)	*Prangos ferulacea*
O. kurdica	MT026611	MT027286			Armenia	Garni, 24 May 2016, *R. Piwowarczyk* (ERCB, KTC)	*Prangos ferulacea*
O. laxissima	MT026643	MT027290			Georgia	Untsa, 12 July 2015, *R. Piwowarczyk* (KTC)	*Robinia pseudoacacia*
O. laxissima	MT026664	MT027293			Armenia	Lichk, 17 June 2017, *R. Piwowarczyk* (ERCB, KTC)	*Fraxinus excelsior*
O. laxissima	KR260916	MT027291			Georgia	Sighnaghi, 28 May 2014, *R. Piwowarczyk* (KTC)	*Carpinus*
O. laxissima	KR260927	MT027300			Azerbaijan	Gabala Hanlar, 9 June 2012, *L.I. Krupkina, B.I. Tatanov, B.B. Svanova* (LE)	*Carpinus*
O. laxissima	KR260917	MT027292			Georgia	Antoki S, 29 May 2014, *R. Piwowarczyk* (KTC)	*Fraxinus*
O. laxissima	MN207121				Georgia	Sighnaghi, 16 May 2019, *R. Piwowarczyk* (KTC)	*Punica granatum*
O. leptantha (O. icterica)	KY513970*	KY484473*			Spain	Piwowarczyk et al. 2018d	*Centaurea*

O. lucorum	KY218641*	KY484491*			Poland	Piwowarczyk et al. 2017a / Piwowarczyk et al. 2018d	*Berberis vulgaris*
O. lutea	KY218650*	KY484467*			Poland	Piwowarczyk et al. 2017a / Piwowarczyk et al. 2018d	*Medicago sativa*, *M. falcata*
O. lutea	KY513967*	KY484468*			Poland	Piwowarczyk et al. 2018d	*Medicago sativa*
O. lutea	KY218655*	MT027303			Armenia	Piwowarczyk et al. 2017a / Byuryakan, Aragats Mt, 8 July 2015, *R. Piwowarczyk* (KTC)	*Medicago sativa*
O. lutea	KY218656*	MT027302			Armenia	Piwowarczyk et al. 2017a / Lusaghbyur – Lernut, 11 July 2015, *R. Piwowarczyk* (KTC)	*Medicago sativa*
O. lutea	AY209279*				Georgia	Schneeweiss et al. 2004	*Medicago*
O. lycoctoni	KY218660*	MG987215*			Switzerland	Piwowarczyk et al. 2017a / Piwowarczyk et al. 2018b	*Aconitum lycoctonum*
O. lycoctoni	EU655620*				Spain	Unpublished	*Aconitum*
O. lycoctoni	AY960724*				Switzerland	Carlón et al. 2005	*Aconitum lycoctonum*
O. lycoctoni	EU817099*				Slovenia	Unpublished	*Aconitum*
O. mayeri	KY513951*	KY484489*			Poland	Piwowarczyk et al. 2018d	*Laserpitium latifolium*
O. mayeri	KY513934*	KY484487*			Germany	Piwowarczyk et al. 2018d	*Laserpitium latifolium*
O. minor	KR260923	KY484485*			Poland	Ułanica, 31 June 2014, *M. Wolanin* (KTC) / Piwowarczyk et al. 2018d	*Trifolium pratense*
O. minor	KR260918	MT027288			Georgia	Kortaneti, 31 May 2014, *R. Piwowarczyk* (KTC)	*Lactuca*
O. minor	KR260919	MT027289			Georgia	Dviri, 2 June 2014, *R. Piwowarczyk* (KTC)	*Chondrilla*
O. minor	AF437315*					Unpublished	
O. minor			AY348568*			Trakulnaleamsai et al. 2005	
O. minor				KC542249*		McNeal et al. 2013	
O. mlokosiewiczii	KY218654*	MG987211*			Georgia	Piwowarczyk et al. 2017a / Piwowarczyk et al. 2018b	*Aconitum cymbulatum*
O. mlokosiewiczii		MT027313			Georgia	Gveleti, Great Caucasus, 18 July 2015, *R. Piwowarczyk* (KTC)	*Aconitum cymbulatum*
O. owerinii	MT026672	MT027295			Armenia	Ghazanchi, 22 July 2017, *R. Piwowarczyk* (KTC)	*Trifolium trichocephalum*

O. owerinii	MT026669	MT027294			Armenia	between Ardenis and Aghvorik, 22 July 2017, *R. Piwowarczyk* (ERCB, KTC)	*Vicia iberica*
O. owerinii	MT026668	MT027299			Armenia	between Ardenis and Aghvorik, 22 July 2017, *R. Piwowarczyk* (ERCB, KTC)	*Vicia iberica*
O. owerinii	MT026642	MT027297			Georgia	SW of Vale, 14 July 2015, *R. Piwowarczyk* (KTC)	*Trifolium*
O. owerinii	MT026665	MT027298			Armenia	Ghazanchi, 22 July 2017, *R. Piwowarczyk* (KTC)	*Trifolium*
O. owerinii	KT819124	MT027296			Georgia	Gudauri, 16 July 2015, *R. Piwowarczyk* (KTC)	*Trifolium canescens*
O. pallidiflora	KY513960*	KY484499*			Poland	Piwowarczyk et al. 2018d	*Cirsium oleraceum*
O. pancicii		KT387724*				Cusimano and Wicke 2016	
O. pancicii	JN796923*				Austria	Piednoël et al. 2012	*Knautia drymeia*
O. picridis	KR260922*	KY484486*			Poland	Piwowarczyk et al. 2018d	*Picris hieracioides*
O. picridis	KR260920*	KU238867*			Poland	Piwowarczyk et al. 2017a / Kwolek et al. 2017	*Picris hieracioides*
O. pubescens	AY209268*				Greece	Schneeweiss et al. 2004	
O. pycnostachya	AY881143*					Unpublished	
O. raddeana	MT026654	MT027323			Georgia	Adjara, Mlashe, 13 July 2015, *R. Piwowarczyk* (KTC)	*Campanula*
O. raddeana	AY209258*				Georgia	Schneeweiss et al. 2004	*Campanula*
O. raddeana	KY218657*				Georgia	Piwowarczyk et al. 2017a	*Asyneuma*
O. raddeana	AY209257*				Georgia	Schneeweiss et al. 2004	*Campanula*
O. raddeana	MT026644	MT027324			Georgia	Jvari pass, near Gudauri, 19 July 2015, *R. Piwowarczyk* (KTC)	*Campanula*
O. raddeana			AM233995*			Bennett and Mathews 2006	
O. raddeana				KC542250*		McNeal et al. 2013	
O. rapum-genistae		MF964232*			Spain	Piwowarczyk et al. 2018d	*Cytisus*
O. rapum-genistae	AY209280*				France	Schneeweiss et al. 2004	

O. reticulata	KY513957*	KY484501*			Slovakia	Piwowarczyk et al. 2018d	*Carduus glaucinus*
O. rigens	MT026587				Italy	Sardinia, *R. Piwowarczyk* (KTC)	*Genista*
O. salviae	AY209252*				Austria	Schneeweiss et al. 2004	*Salvia glutinosa*
O. sanguinea (O. crinita)	AY209244*				Italy	Schneeweiss et al. 2004	
O. santolinae	EU655604				Spain	Carlón et al. 2008	
O. schelkovnikovii	MT026671	MT027304			Armenia	Semyonovka, 26 July 2017, *R. Piwowarczyk* (ERCB, KTC)	*Cirsium leucocephalum*
O. schelkovnikovii	MT026670	MT027305			Armenia	Norabak, 25 July 2017, *R. Piwowarczyk* (KTC)	*Cirsium schelkovnikovii*
O. schelkovnikovii	MT026597	MT027306			Armenia	S of Vardenik, 28 July 2017, *R. Piwowarczyk* (ERCB, KTC)	*Cirsium aduncum*
O. serbica (O. ozanonis)	AY960723*				France	Carlón et al. 2005	
O. sintenisii	AY209276*				Turkey	Schneeweiss et al. 2004	*Prangos ferulacea*
O. teucrii	KR260924*	KY484464*			Austria	Piwowarczyk et al. 2018d	*Teucrium montanum*
O. transcaucasica	AY209263*				Turkey	Schneeweiss et al. 2004	
O. transcaucasica	AY209261*				Turkey	Schneeweiss et al. 2004	*Rhus coriaria*
O. zajaciorum	MT026633	MT027322			Georgia	between Tsaghveri and Kimotesubani, 2 June 2014, *R. Piwowarczyk* (KTC)	*Scutellaria sosnowskyi*
Phacellanthus							
P. tubiflorus	KY706615*		KY706630*	KY706632*		Fu et al. 2017	
Phelipanche							
P. aegyptiaca	MG948171*				Iran	Unpublished	
P. aegyptiaca	KC811171*				China	Unpublished	
P. arenaria	KY513940*	KU238868*			Poland	Piwowarczyk et al. 2018d / Kwolek et al. 2017	*Artemisia campestris*
P. arenaria	KY513949*	KY484508*			Czech Republic	Piwowarczyk et al. 2018d	*Artemisia campestris*
P. arenaria	MT026651	MT027332			Armenia	between Semyonovka and Tsovagyugh, 9 July 2015, *R. Piwowarczyk* (KTC)	*Artemisia campestris* subsp.*inodora*

P. arenaria	MT026636	MT027333			Georgia	between Rustavi and Idumala, 2014, *R. Piwowarczyk* (KTC)	*Artemisia*
P. bohemica	KY513953*	KU238872*			Poland	Piwowarczyk et al. 2018d / Kwolek et al. 2017	*Artemisia campestris*
P. bohemica	KY513965*	KY484504*			Poland	Piwowarczyk et al. 2018d	*Artemisia campestris*
P. caesia	KY513936*	KY484506*			Ukraine	Piwowarczyk et al. 2018d	*Artemisia austriaca*
P. caesia	MT026667	MT027334			Armenia	Ghazanchi, 22 July 2017, *R. Piwowarczyk* (ERCB, KTC)	*Artemisia armeniaca*
P. caesia	MT026657	MT027335			Armenia	between Karmrashen and Goghtanik, 20 June 2017, *R. Piwowarczyk* (ERCB, KTC)	*Artemisia absinthium*
P. caesia	MT026612	MT027337			Armenia	Gavar SE, 10 June 2016, *R. Piwowarczyk* (KTC)	*Artemisia fragrans*
P. caesia	MT026621	MT027336			Armenia	Noratus SE, 10 June 2016, *R. Piwowarczyk* (KTC)	*Artemisia incana*
P. cernua (P. inexpectata)	AY960739*				Spain	Carlón et al. 2005	*Lactuca virosa*
P. cernua	MT026591	MT027345			Spain	Merindad del Río Ubierna, 2007, herb. Ó. Sánchez Pedraja	*Lactuca viminea*
P. cernua	MT026673	MT027346			Armenia	Nerkin Giratagh, 15 June 2017, *R. Piwowarczyk* (ERCB, KTC)	*Lactuca wilhelmsiana*
P. cilicica	MT026629	MT027351			Armenia	Vayk – Saravan, 27 May 2016, *R. Piwowarczyk* (KTC)	*Phlomis orientalis*
P. cilicica	MT026623	MT027353			Armenia	Azat Reservoir near Lanjazat, 25 May 2016, *R. Piwowarczyk* (KTC)	*Stachys inflata*
P. cilicica	MT026613	MT027352			Armenia	Zaritap, 31 May 2016, *R. Piwowarczyk* (KTC)	*Phlomis orientalis*
P. coelestis	MT026677	MT027273			Armenia	between Karmrashen and Goghtanik, 20 June 2017, *R. Piwowarczyk* (ERCB, KTC)	*Silene spergulifolia*
P. coelestis	MT026662	MT027277			Armenia	Lichk – Chgnavor Mt, 17 July 2017, *R. Piwowarczyk* (KTC)	*Silene*
P. coelestis	MT026652				Armenia	between Semyonovka and Tsovagyugh, 9 July 2015, *R. Piwowarczyk* (KTC)	*Asperula glomerata*
P. coelestis	MT026616	MT027274			Armenia	between Kajaran and Tashtun pass, 3 June 2016, *R. Piwowarczyk* (KTC)	*Silene iberica*
P. bungeana	MT026661				Armenia	Kapan SE, 18 June 2017, *R. Piwowarczyk* (ERCB, KTC)	*Teucrium chamaedrys*
P. bungeana	MT026647	MT027339			Armenia	Lusaghbyur E, 11 July 2015, *R. Piwowarczyk* (KTC)	*Teucrium chamaedrys*
P. georgii-reuteri	EU581800*				Spain	Carlón et al. 2008	*Lepidium subulatum*
P. georgii-reuteri	AY960746*				Spain	Carlón et al. 2005	*Lepidium subulatum*

P. gratiosa	EU581791*				Spain	Carlón et al. 2008	*Launea arborescens*
P. hajastanica	MT026614	MT027354			Armenia	Vayk SE, 31 May 2016, *R. Piwowarczyk* (ERCB, KTC)	*Nepeta trautvetteri*
P. heldreichii	MT026624	MT027280			Armenia	Khachik, 29 May 2016, *R. Piwowarczyk* (KTC)	*Eryngium campestre*
P. iberica	AY960742*				Turkey	Carlón et al. 2005	
P. lavandulacea	MT026585	MT027272			Montenegro	Valdanos near Ulcinj, 2 May 2016, *R. Piwowarczyk* (KTC)	*Aspalthium bituminosum*
P. libanotica	MT026626	MT027278			Armenia	Artavan, 27 May 2016, *R. Piwowarczyk* (KTC)	*Prunus fenzliana*
P. libanotica (O. orientalis)	MT026584	MT027279			Armenia	Meghri N, 28 June 1956, *T.E. Egorova* et al. (LE)	*Prunus*
P. mutellii	AY209340*				Spain	Schneeweiss et al. 2004	
P. mutellii	AY209341*				Italy	Schneeweiss et al. 2004	
P. nana	EU581808*				Spain	Carlón et al. 2008	
P. nana	AY209313*				Greece	Schneeweiss et al. 2004	
P. nana	AY209312*				Italy	Schneeweiss et al. 2004	*Oxalis pes-caprae*
P. nana	EU581804*				France	Carlón et al. 2008	
P. nana	MT026625	MT027269			Armenia	Dilijan W, 9 June 2016, *R. Piwowarczyk* (KTC)	*Caucalis, Medicago*
P. nowackiana	AY209352*				Greece	Schneeweiss et al. 2004	*Alyssum* cf. *heldreichii*
P. oxyloba	AY209319*				Greece	Schneeweiss et al. 2004	
P. oxyloba	AY209321*				Turkey	Schneeweiss et al. 2004	
P. portoilicitana	MT026679	MT027347			Armenia	Lanjanist SW, 13 June 2017, *R. Piwowarczyk* (KTC)	*Centaurea behen*
P. portoilicitana	MT026675	MT027349			Armenia	Urtsalanj SE, 14 June 2017, *R. Piwowarczyk* (KTC)	*Centaurea behen*
P. portoilicitana	MT026660	MT027348			Armenia	Lanjanist SW, 13 June 2017, *R. Piwowarczyk* (KTC)	*Centaurea behen*
P. portoilicitana	MT026592	MT027350			Spain	Villarrubia de Santiago, 2013, *L. Carlón & G. Moreno Moral* (herb. Ó. Sánchez Pedraja)	*Centaurea*
P. portoilicitana	AY960743*				Spain	Carlón et al. 2005	*Centaurea*
P. pulchella	AY960741*				Georgia	Carlón et al. 2005	

P. purpurea	MT026610	KY484503*			Poland	Chrzanów, 21 June 2014, *R. Piwowarczyk* (KTC) / Piwowarczyk et al. 2018d	*Achillea millefolium*
P. purpurea	KY513964*	KY484505*			Poland	Piwowarczyk et al. 2018d	*Achillea millefolium*
P. purpurea	MT026648	MT027343			Armenia	Antarut, Aragats Mt, 8 July 2015, *R. Piwowarczyk* (KTC)	*Achillea millefolium*
P. purpurea	MT026649	MT027342			Georgia	Abastumani N, 12 June 2015, *R. Piwowarczyk* (KTC)	*Tripleurospermum*
P. purpurea	MT026634	MT027340			Georgia	Kimotesubani, 2 June 2014, *R. Piwowarczyk* (KTC)	*Tanacetum*
P. purpurea	MT026650	MT027341			Armenia	between Aznvadzor and Puskhin Pass, 10 July 2015, *R. Piwowarczyk* (KTC)	*Achillea*
P. ramosa	KY513946*	KY484502*			Poland	Piwowarczyk et al. 2018d	*Nicotiana tabacum*
P. ramosa	KY513945*	KU238870*			Poland	Piwowarczyk et al. 2018d / Kwolek et al. 2017	*Lycopersicon esculentum*
P. ramosa	EU581794*				Spain	Carlón et al. 2008	
P. ramosa			AM233996*			Bennett and Mathews 2006	
P. reuteriana (P. tunetana)	AY209325*				Spain	Schneeweiss et al. 2004	*Plantago albicans*
P. rosmarina	EU581736*				Portugal	Carlón et al. 2008	*Rosmarinus officinalis*
P. rosmarina	EU581735*				Spain	Carlón et al. 2008	*Rosmarinus officinalis*
P. schultzii	MT026593	MT027282			Spain	Albox, 2011, *G. Moreno Moral* (herb. Ó. Sánchez Pedraja)	*Ferula communis*
P. sevanensis	MT026646	MT027281			Armenia	Tsovagyugh E, 9 July 2015, *R. Piwowarczyk* (KTC)	*Heracleum trachyloma*
P. zangezuri	MT026615	MT027338			Armenia	between Kajaran and Lichk, 3 June 2016, *R. Piwowarczyk* (ERCB, KTC)	*Tragopogon pterocarpus*
P. sp. on *Artemisia*	MT026617				Armenia	Zaritap, 31 May 2016, *R. Piwowarczyk* (KTC)	*Artemisia*
P. sp. on *Alyssum*	MT026635	MT027264			Georgia	between Rustavi and Idumala, 2014, *R. Piwowarczyk* (KTC)	*Alyssum*
P. sp. on *Astrodaucus*	MT026618	MT027275			Armenia	Tashtun E, 3 June 2016, *R. Piwowarczyk* (KTC)	*Astrodaucus*
P. sp. on *Astrodaucus*	MT026658	MT027276			Armenia	Lichk – Chgnavor Mt, 17 July 2017, *R. Piwowarczyk* (KTC)	*Astrodaucus*
P. sp. on *Genista*	MT026666	MT027270			Armenia	Kapan, 18 June 2017, *R. Piwowarczyk* (KTC)	*Genista*
P. sp. on *Melilotus*	MT026674	MT027271			Armenia	Jrapi SE, 11 June 2017, *R. Piwowarczyk* (KTC)	*Melilotus officinalis*
P. sp. on *Nonea*	MT026676	MT027266			Armenia	Shikahogh N, 18 June 2017, *R. Piwowarczyk* (KTC)	*Nonea lutea*
P. sp. on *Xeranthemum*	MT026659	MT027268			Armenia	Nerkin Giratagh, 15 June 2017, *R. Piwowarczyk* (KTC)	*Xeranthemum*

Phelypaea							
P. boissieri	MT026607				N. Macedonia	Kozjak, near Trojaci, in the vicinity of the town Prilep, 29 May 2019, *Z. Nikolov* (HMMNH)	*Centaurea grbavacensis*
P. coccinea	MT026627				Armenia	Fantan, 6 June 2016, *R. Piwowarczyk* (KTC)	*Centaurea pseudoscabiosa*
P. coccinea	MT026638	MT027344			Georgia	Davit Gareja, 26 May 2014, *R. Piwowarczyk* (KTC)	*Psephellus*
P. coccinea	MT026678				Armenia	Jermuk, 22 June 2017, *R. Piwowarczyk* (KTC)	*Klasea coriacea*
P. coccinea	MT026680				Armenia	Saralanj, 14 June 2017, *R. Piwowarczyk* (KTC)	*Centaurea pseudoscabiosa*
P. tournefortii	MT026628				Armenia	Khosrov, 24 May 2016, *R. Piwowarczyk* (KTC)	*Tanacetum polycephalum*
P. tournefortii	MT026630				Armenia	Garni, 25 May 2016, *R. Piwowarczyk* (KTC)	*Tanacetum polycephalum*
P. tournefortii	AY209285*				Turkey	Schneeweiss et al. 2004	*Tanacetum*

Table S1 (Supplementary Materials) List of taxa and sequences analysed (* sequences obtained from GenBank)
