# Supplementary figures and images for: Phylogeny and historical biogeography analysis support Caucasian and Mediterranean centres of origin of key holoparasitic Orobancheae (Orobanchaceae) lineages
Source: PhytoKeys. 2021 Mar 12;174:165–94. doi: 10.3897/phytokeys.174.62524 (PMC7979677; doi:10.3897/phytokeys.174.62524)

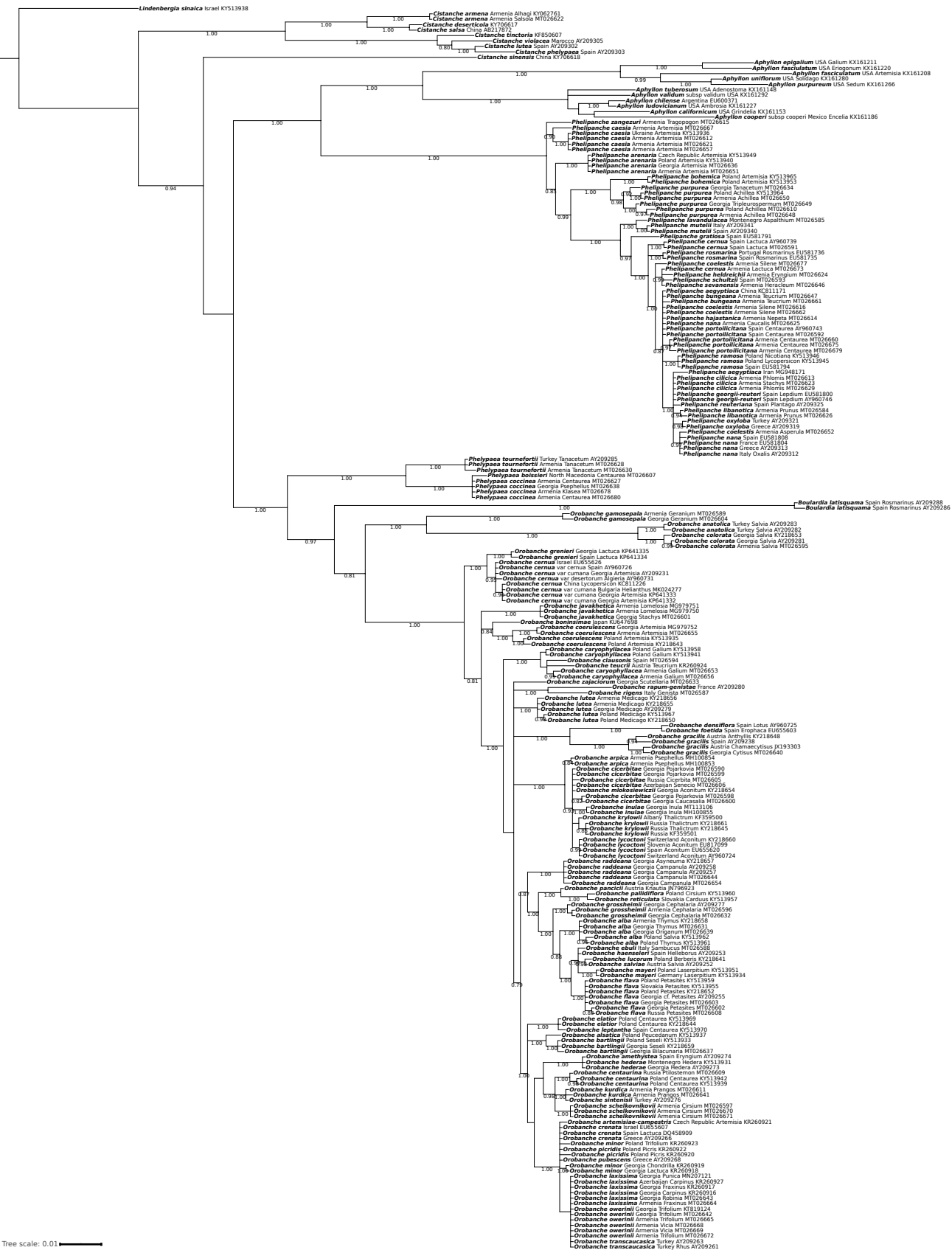

Supplement: Supplementary material 2 — Figure S1 [file phytokeys-174-165-s002.pdf]

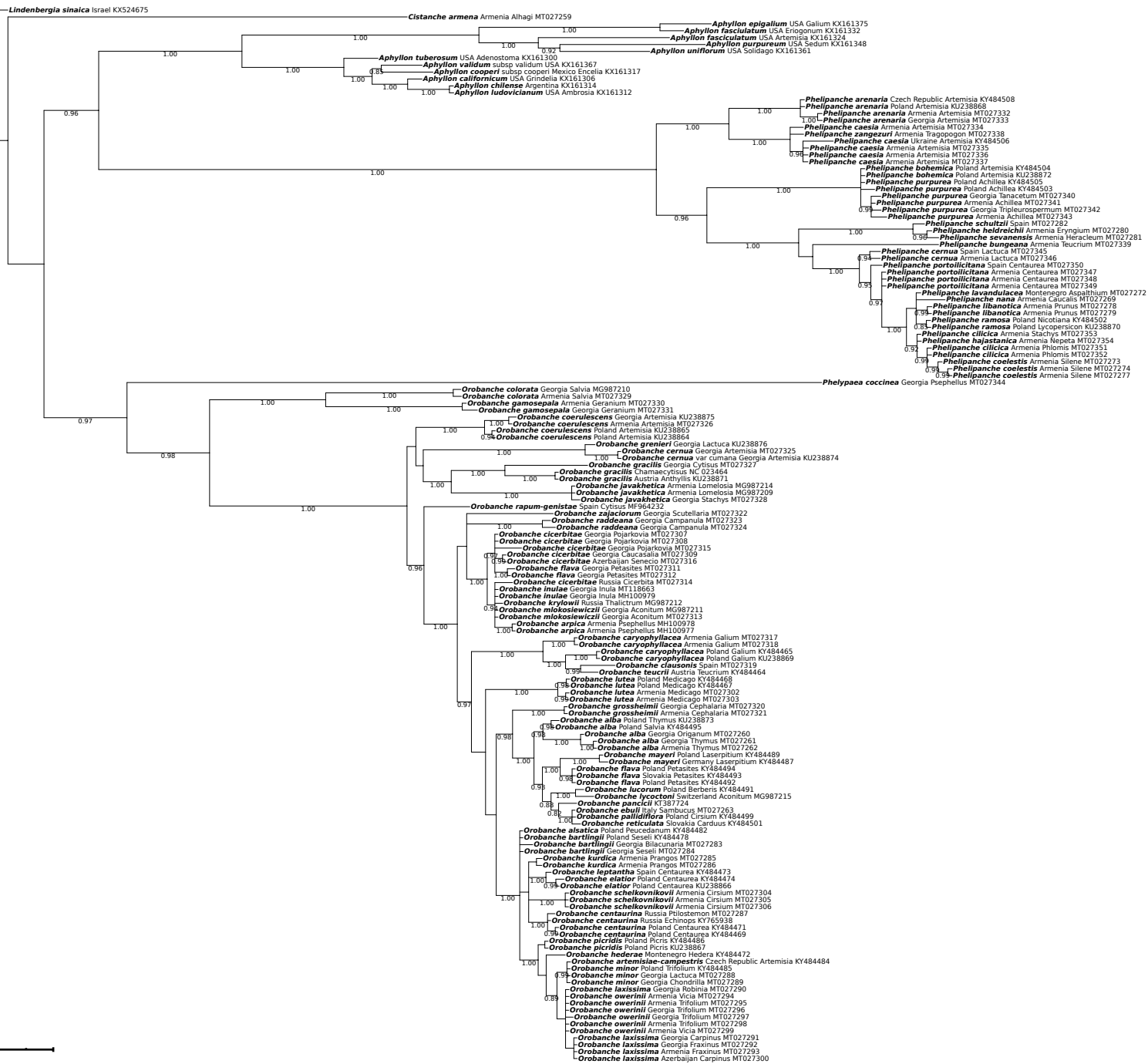

Supplement: Supplementary material 3 — Figure S2 [file phytokeys-174-165-s003.pdf]
